# Quantifying the impact of interventions against *Plasmodium vivax*: A model for country-specific use

**DOI:** 10.1016/j.epidem.2024.100747

**Published:** 2024-03

**Authors:** C. Champagne, M. Gerhards, J.T. Lana, A. Le Menach, E. Pothin

**Affiliations:** a Swiss Tropical and Public Health Institute, Basel, Switzerland; b University of Basel, Basel, Switzerland; c Clinton Health Access Initiative, Boston, USA

**Keywords:** Plasmodium vivax model, Malaria model, Plasmodium vivax intervention

## Abstract

In order to evaluate the impact of various intervention strategies on *Plasmodium vivax* dynamics in low endemicity settings without significant seasonal pattern, we introduce a simple mathematical model that can be easily adapted to reported case numbers similar to that collected by surveillance systems in various countries. The model includes case management, vector control, mass drug administration and reactive case detection interventions and is implemented in both deterministic and stochastic frameworks. It is available as an R package to enable users to calibrate and simulate it with their own data. Although we only illustrate its use on fictitious data, by simulating and comparing the impact of various intervention combinations on malaria risk and burden, this model could be a useful tool for strategic planning, implementation and resource mobilization.

## Highlights

• A model for Plasmodium vivax malaria including control interventions is presented.
• It includes treatment, reactive case detection, mass chemoprevention, vector control.
• The model is publicly available as an R package.
• Practical applications are illustrated on a simulated example with fictitious data.
• It could support country strategic planning, implementation or resource mobilization.

## 1 Introduction

*Plasmodium vivax* malaria is a parasitic infection responsible for about 4.9 million cases in 2021 (World Health Organization, 2022b). While generally considered as less severe than *Plasmodium falciparum* malaria, *P. vivax* represents the majority of the remaining cases in many countries where elimination goals have been set for 2025 or 2030, including many countries in Asia (Bhutan, Nepal, Thailand, Republic of Korea, Democratic People’s Republic of Korea), the Pacific (Vanuatu) and the Americas (Costa Rica, Ecuador, Guatemala, Honduras, Mexico, Panama, Suriname) (World Health Organization, 2022b, World Health Organization, 2021). Due to various distinguishing biological characteristics such as its capacity to remain dormant in the liver of infected individuals before its reactivation or its very early transmissibility potential, *P. vivax* is particularly difficult to eliminate. Therefore, reaching the elimination targets requires specific strategies that combine the various available interventions. These interventions include anti-malarial treatments, chemoprevention, case detection and vector control (World Health Organization et al., 2015a, Bassat et al., 2016) and are described in Table 1, highlighting their specific strengths and weaknesses.

Because of the advantages and challenges involved with each type of intervention, defining the most appropriate combination of interventions highly depends on context-specific factors, such as the level of transmission, ecological factors, the vulnerability to case importation or the characteristics of the health system (Cohen et al., 2017). Therefore, the choice and adaptation of the malaria intervention strategy required to reach elimination targets needs to be tailored to local context. Mathematical modelling can be used to quantify the impact of the considered intervention strategies, identify the most impactful ones in each setting (Owen et al., 2022). Various *P. vivax* models have recently been developed with increasing level of details in the depiction of biological processes and interventions (Anwar et al., 2023b). For instance, the impact of interventions such as vector control (White et al., 2018), mass chemoprevention (Robinson et al., 2015, Obadia et al., 2022, Anwar et al., 2023a) and case management (Chamchod and Beier, 2013, Nekkab et al., 2021) also considering the impact of treatment delays (Kim et al., 2021) have been modelled at the population level. Nonetheless, few models allow for all interventions to be included simultaneously (White et al., 2018, Silal et al., 2019, Tian et al., 2022) and they do not incorporate reactive case detection. Additionally, the most detailed models are not always readily operationalized for being used routinely at country level because their calibration to routine data is not straightforward to implement, especially in the presence of external importation.

**Table 1:** Description of the considered interventions.

Intervention and mode of action	Advantages	Limitations
**Treatment/Case management**:	• Reduces disease burden (Chu and White, 2021)	• 8-aminoquinolines have restricted eligibility criteria, long treatment scheme and potential adverse effects, especially in patients with glucose-6-phosphate dehydrogenase (G6PD) deficiency (Bassat et al., 2016, Chu and White, 2021, Thriemer et al., 2021)
• Clearance of blood-stage parasites responsible for the acute infection	• Reduces the parasite’s capacity for ongoing transmission by reducing the number of relapses and the number of infectious days (Chu and White, 2021)	• Reaching high effective treatment coverage depends on various components, including physical accessibility to diagnostic and treatment, compliance of the health system or adherence to the treatment scheme (Galactionova et al., 2015, Emerson et al., 2022)
• Clearance of dormant liver-stage parasites (radical cure) with 8-aminoquinolines such as primaquine (PQ) and tafenoquine (TQ)		• The effectiveness on transmission is limited given the high proportion of patients presenting to health facilities with gametocytes (Douglas et al., 2010) indicating they might already have participated in transmission before being treated.

**MDA**: Mass drug administration or mass chemoprevention	• A potential elimination accelerator permitting a rapid reduction of the parasite reservoir in the host population	• The evidence from randomized control trials points to a positive short term effect that is most likely not sustained in the longer term if elimination is not reached or if not complemented with additional interventions used in combination (Poirot et al., 2013, Shah et al., 2021).
	• Deployed in the past in various settings with various success rates (Poirot et al., 2013)	• Logistical, safety and acceptability challenges, especially regarding the mass use of 8-aminoquinolines
		• Mass use of 8-aminoquinolines not recommended by the World Health Organization (World Health Organization et al., 2015a, Alonso, 2020, World Health Organization, 2022a)

**Reactive case detection**: Typically, investigations are conducted in the vicinity of index cases so to enhance the likelihood of finding additional infections that would otherwise have escaped the health system	• Reduces community transmission by identifying infected individuals that have yet to (and may not plan to) seek care	• Challenges in the feasibility of this resource intensive activity which may take resources away from routine surveillance and diagnosis activities
	• Part of the WHO strategic pillar to “transform malaria surveillance into a core intervention” (World Health Organization et al., 2015b)	• Uncertainty in the conditions for its effectiveness and difficulty to monitor effectiveness (van Eijk et al., 2016, Perera et al., 2020, Hetzel and Chitnis, 2020)
	• Credited to contributing to the successful elimination strategies in China and El Salvador (Perera et al., 2020, Burton et al., 2018, Balakrishnan, 2021)	

**Vector control**: Killing or repelling the *Anopheles* mosquitoes responsible for parasite transmissions, via interventions such as insecticide treated bednets (ITNs) or indoor residual spraying (IRS)	• Complementary elimination tool to reduce the likelihood of transmission when cases are not treated promptly or correctly	• Many eliminations settings outside of Africa harbour mosquitoes species with different characteristics, such as early biting behaviours or different response to insecticides that reduce intervention effectiveness (Briët et al., 2019, Monroe et al., 2020).
	• Key component of the WHO recommended prevention strategies (World Health Organization et al., 2015a, World Health Organization, 2023)	
	• ITNs has been one of the most important drivers of the reductions in malaria burden in Africa in the beginning of the 21st century (Bhatt et al., 2015)	• Logistical challenges to optimize deployment timing given the limited duration of effectiveness (especially IRS)
		• Acceptability and logistical challenges to reaching high coverage/usage of the interventions
		• Short term durability of the insecticides and nets, requiring frequent re-deployment of the interventions

In order to address these shortcomings, a model was previously developed to represent *P. vivax* dynamics in low endemicity settings without strong seasonality (Champagne et al., 2022). Building on White et al. (2016), this simple compartmental model includes case management interventions, external importation and a methodology to infer parameter values from available data based on the steady-state assumption (Champagne et al., 2022). Nonetheless, this model has some limitations that restrict its use in practice. Firstly, the model assumes that treatment acts instantaneously, such that treated patients do not contribute to ongoing disease transmission. This assumption is not totally appropriate for *P. vivax*, for which treated patients might already have participated in transmission before the effect of their treatment (Douglas et al., 2010). Secondly, it does not include other interventions such as reactive case detection (RCD) or mass drug administration (MDA), which are part of the malaria elimination toolbox for decision-makers. Finally, it is only implemented in the deterministic framework.

The present work therefore extends the model by Champagne et al. (2022) by removing these limitations, thus increasing its potential applications for country-specific decision making. Such use-cases are illustrated in an example on three fictitious areas of varying endemicity. The extended model is publicly available as an R package (https://swisstph.github.io/VivaxModelR/) that can be applied to the users’ own data.

## 2 Methods

### 2.1 Model of *P. vivax* dynamics with delay in treatment effect (model 1)

*P. vivax* dynamics are represented by a compartmental model where the host population is divided between infectious and susceptible individuals who do or do not harbour liver stage parasites (White et al., 2016, Champagne et al., 2022). In order to allow the possibility for treated individuals to infect mosquitoes before they effectively clear their parasites, the model by Champagne et al. (2022) is modified by adding two compartments representing treated individuals. Let $T_{L}$ be the proportion of blood-stage infected population with liver-stage infection which receive effective treatment, $T_{0}$ that of blood-stage infected population without liver-stage infection which receive effective treatment, $U_{L}$ that of blood-stage infected population with liver-stage infection but which do not receive effective treatment and $U_{0}$ that of blood-stage infected population without liver-stage infection which do not receive effective treatment. We define $I≔T_{L}+T_{0}+U_{L}+U_{0}$ as the proportion of blood-stage infections. $S_{0}$ represents the proportion of fully susceptible individuals, and $S_{L}$ represents the individuals who have cleared their blood stage parasites but still harbour liver stage parasites and hence have the possibility to experience a relapse. The model can be represented by the following system of equations:

(1) $$\frac{dU_{L}}{dt}=\left(1-\alpha \right)\left(\lambda I+\delta \right)\left(S_{0}+S_{L}\right)+\left(\lambda I+\delta \right)U_{0}+\left(1-\alpha \right)fS_{L}-\gamma_{L}U_{L}$$

$$\;-rU_{L}$$

$$\frac{dU_{0}}{dt}=-\left(\lambda I+\delta \right)U_{0}+\gamma_{L}U_{L}-rU_{0}$$

$$\frac{dT_{L}}{dt}=\alpha \left(\lambda I+\delta \right)\left(S_{0}+S_{L}\right)+\alpha fS_{L}-\left(\sigma +r+\gamma_{L}\right)T_{L}+\left(\lambda I+\delta \right)T_{0}$$

$$\frac{dT_{0}}{dt}=\gamma_{L}T_{L}-\sigma T_{0}-\left(\lambda I+\delta \right)T_{0}-rT_{0}$$

$$\frac{dS_{L}}{dt}=\left(1-\beta \right)\sigma T_{L}-\left(\lambda I+\delta \right)S_{L}-fS_{L}-\gamma_{L}S_{L}+rU_{L}+rT_{L}$$

$$\frac{dS_{0}}{dt}=-\left(\lambda I+\delta \right)S_{0}+\beta \sigma T_{L}+\sigma T_{0}+\gamma_{L}S_{L}+rU_{0}+rT_{0}$$

**Fig. 1:**
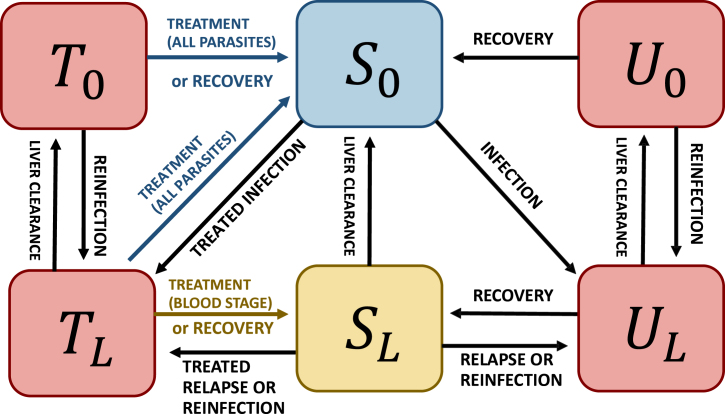
Schematic representation of the model *P. vivax* dynamics including delay in treatment effect (model 1). In red are depicted the infectious compartments, with effective treatment access ($T_{0}$ and $T_{L}$) and without ($U_{0}$ and $U_{L}$), the subscripts L and 0 denoting respectively the presence and absence of liver-stage parasites. Blue indicates malaria-free individuals ($S_{0}$) while yellow corresponds to individuals without blood-stage infection but with latent liver-stage parasites ($S_{L}$).

All other parameter notations are indicated in Table 2 and a schematic representation of the model is presented in Fig. 1. A description of the rationale for choosing the formulation in Eq. (1) is presented in Appendix A in the context of perfect radical cure.

In this model, case management is thus represented via three parameters. The first parameter is the proportion of individuals receiving effective treatment (α), i.e. the proportion of individuals whose blood-stage parasites are effectively cleared due to treatment, if they do not recover naturally before. The second parameter (β) is the proportion of effectively treated individuals who experience radical cure, i.e. the clearance of liver-stage parasites in addition to blood-stage parasites. The third parameter (1/σ) quantifies the time during which an effectively treated individual can transmit the disease to mosquitoes (ignoring potential recoveries that could happen during this interval, see Appendix A for more details). All three parameters can be informed by data on the health system, following the effective coverage framework (Galactionova et al., 2015). For example, the parameters α and β can be informed by a decision tree accounting for access to care, testing, compliance, adherence, drug eligibility and drug efficacy, similar to Galactionova et al. (2015) or White et al. (2018), in order to represent a realistic treatment pathway. The parameter 1/σ can represent the sum of three components, namely the time from onset of infectiousness to symptoms, the time to access the correct treatment within healthcare and the time for the drug to prevent onward transmission.

In this model, the only difference between treated ($T_{L}$ and $T_{0}$) and untreated ($U_{L}$ and $U_{0}$) individuals is the duration of their infectiousness. The distinction between treated and untreated is represented at the time of infection, but this is equivalent to distinguishing them at the time treatment is effective, as shown in Appendix A. As long as the parameter α is a realistic estimate of effective care coverage, the interpretation in terms of treatment pathway can be made.

Additionally, we define the observed incidence $h≔\rho \left[\left(\lambda I^{*}+\delta \right)\left(1-I^{*}\right)+fS_{L}^{*}\right]$ as the rate of observed newly arising blood-stage infections, where $I^{*}$ and $S_{L}^{*}$ are the equilibrium proportions for I and $S_{L}$ respectively and ρ is a reporting rate (proportion of infections that are effectively reported in the data). Similarly to the case management parameters α and β, the parameter ρ can be informed by a decision tree to reflect the pathway of case reporting in the surveillance system. We also define the proportion of imported cases p such that $ph=\rho \delta \left(1-I^{*}\right)$.

The corresponding reproduction numbers in the presence of control interventions ($R_{c}$) and in the absence of control ($R_{0}$) are calculated using the next-generation matrix approach (van den Driessche and Watmough, 2002) as follows:

$$R_{c}=\frac{\lambda \left(f+\gamma_{L}\right)\left(\gamma_{L}+r\right)\left(\gamma_{L}+r+\sigma \right)\left(r+\sigma \left(1-\alpha \right)\right)}{r\left(r+\sigma \right)\left(\gamma_{L}\left(f+\gamma_{L}+r\right)\left(\gamma_{L}+r+\sigma \right)+\alpha f\sigma \left[\beta \left(r+\gamma_{L}\right)-\gamma_{L}\right]\right)}$$

and

$$R_{0}=\frac{\lambda \left(f+\gamma_{L}\right)\left(\gamma_{L}+r\right)}{r\gamma_{L}\left(f+\gamma_{L}+r\right)}$$

As expected, the basic reproduction number $R_{0}$ in the absence of control is identical to the one in the model where the delay in treatment effect was neglected (Champagne et al., 2022), because in this hypothetical context no individual receives any treatment. Similarly to Champagne et al. (2022), a polynomial relationship between the transmission rate λ and observable quantities h and p can be calculated, as detailed in Appendix B. With this relationship, the model can easily be calibrated to reported incidence data.

**Table 2:** Description of state variables and model parameters.

Notation	Description	Unit	Definition range
**State variables**			
$U_{L}$	Untreated individuals with liver and blood stage parasites	.	[0,1]
$U_{0}$	Untreated individuals with blood stage parasites only	.	[0,1]
$S_{L}$	Susceptible individuals with liver stage parasites	.	[0,1]
$S_{0}$	Fully susceptible individuals	.	[0,1]
$T_{L}$	Treated individuals with liver and blood stage parasites	.	[0,1]
$T_{0}$	Treated individuals with blood stage parasites only	.	[0,1]

**Parameters**			
λ	Transmission rate	time^−1^	≥0
r	Blood stage clearance rate	time^−1^	≥0
$\gamma_{L}$	Liver stage clearance rate	time^−1^	≥0
f	Relapse frequency	time^−1^	≥0
δ	Importation rate	time^−1^	≥0
α	Proportion of infections receiving effective treatment of blood stage parasites	.	[0,1]
β	Proportion of treated infections receiving effective radical cure	.	[0,1]
σ	Inverse of the duration of infectivity for treated infections	time^−1^	≥0
ρ	Observation rate (proportion of new infections that are effectively reported)	.	[0,1]

**RCD model**			
$\iota_{max}$	Maximal number of index cases investigated	time^−1^	≥0
ν	Number of individuals investigated per index case	.	≥0
η	Proportion of effective care for infections detected by RCD	.	[0,1]
τ	Targeting ratio	.	≥0
$\rho_{2}$	Proportion of new reactively-detected infections that are effectively reported	.	[0,1]

**MDA model**			
$c_{MDA}$	MDA coverage	.	[0,1]
$\beta_{MDA}$	Proportion of effective radical cure among individuals receiving MDA	.	[0,1]
$t_{MDA}$	Starting date of MDA	time	≥0
$p_{MDA}$	Duration of MDA prophylaxis	time	≥0

### 2.2 Including reactive case detection (RCD, model 2)

**Fig. 2:**
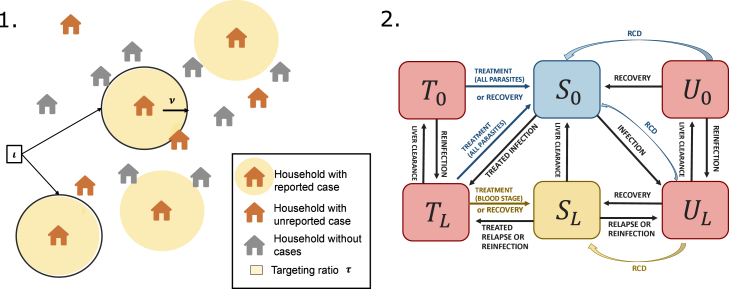
Models including reactive case detection (RCD). 1. Description of the assumptions for modelling RCD. 2. Schematic representation of the RCD model (model (2)). In red are depicted the infectious compartments, with effective treatment access ($T_{0}$ and $T_{L}$) and without ($U_{0}$ and $U_{L}$), the subscripts L and 0 denoting respectively the presence and absence of liver-stage parasites. Blue indicates malaria-free individuals ($S_{0}$) while yellow corresponds to individuals without blood-stage infection but with latent liver-stage parasites ($S_{L}$).

Reactive case detection (RCD) can be included in the model based on the framework by Chitnis et al. (2019). With this approach, reactive case detection adds the possibility for non-treated cases to be detected and treated. The model relies on the assumption that cases are geographically clustered, such that the probability of finding a case in the vicinity of a reported case is higher than the probability of finding a case in the general population. This increased likeliness of finding cases is modelled via the targeting ratio τ as in Chitnis et al. (2019) and Das et al. (2022). The parameter ι indicates the number of index cases investigated per population per unit of time. The parameter ν indicates the number of secondary individuals investigated per index case. We add the parameter η to reflect the effective cure of RCD-detected individuals (including test sensitivity, compliance, adherence and drug efficacy). A schematic representation of the model is presented in Fig. 2. Cases detected via RCD are assumed to receive effective radical cure with the same probability as other treated infections.

The model with delay in treatment effect and reactive case detection, corresponding to Fig. 2, can be represented by the following equations:

(2) $$\frac{dU_{L}}{dt}=\left(1-\alpha \right)\left(\lambda I+\delta \right)\left(1-I\right)+\left(1-\alpha \right)fS_{L}+\left(\lambda I+\delta \right)U_{0}-\gamma_{L}U_{L}-rU_{L}-\;\min\left(\iota_{max},\rho \left(\lambda I+\delta \right)\left(1-I\right)+\rho fS_{L}\right)\nu \tau \eta U_{L}$$

$$\frac{dU_{0}}{dt}=-\left(\lambda I+\delta \right)U_{0}+\gamma_{L}U_{L}-rU_{0}-\;\min\left(\iota_{max},\rho \left(\lambda I+\delta \right)\left(1-I\right)+\rho fS_{L}\right)\nu \tau \eta U_{0}$$

$$\frac{dT_{L}}{dt}=\alpha \left(\lambda I+\delta \right)\left(1-I\right)+\alpha fS_{L}+\left(\lambda I+\delta \right)T_{0}-\gamma_{L}T_{L}-\left(r+\sigma \right)T_{L}$$

$$\frac{dT_{0}}{dt}=-\left(\lambda I+\delta \right)T_{0}+\gamma_{L}T_{L}-\left(r+\sigma \right)T_{0}$$

$$\frac{dS_{L}}{dt}=-\left(\lambda I+\delta +f\right)S_{L}+\left(1-\beta \right)\sigma T_{L}-\gamma_{L}S_{L}+r\left(T_{L}+U_{L}\right)+\;\left(1-\beta \right)\min\left(\iota_{max},\rho \left(\lambda I+\delta \right)\left(1-I\right)+\rho fS_{L}\right)\nu \tau \eta U_{L}$$

$$\frac{dS_{0}}{dt}=-\left(\lambda I+\delta \right)S_{0}+\beta \sigma T_{L}+\sigma T_{0}+\gamma_{L}S_{L}+r\left(T_{0}+U_{0}\right)+\;\min\left(\iota_{max},\rho \left(\lambda I+\delta \right)\left(1-I\right)+\rho fS_{L}\right)\nu \tau \eta \left(\beta U_{L}+U_{0}\right)$$

The number of index cases investigated is written as $\iota =\min\left(\iota_{max},\rho \left(\lambda I+\delta \right)\left(1-I\right)+\rho fS_{L}\right)$ and this formulation ensures that the number of index cases investigated is never higher than the number of cases detected.

This model makes the assumption that cases detected by RCD are immediately effectively treated (transition from $U_{L}$ or $U_{0}$ to $S_{L}$ or $S_{0}$). In some situations, the cases detected via RCD need to be referred to a health facility for treatment, hence they would experience a delay before being cured. Therefore, another version of the model in which infections detected via RCD enter the $T_{0}$ and $T_{L}$ compartments instead of $S_{0}$ and $S_{L}$ was also developed and is presented in Appendix C2 (called “with referral to health facility” or model 2b).

If ιντη=0, this model reduces to the model (1) without RCD. If τ is fixed (or at least bounded), all of the ‘RCD terms’ are of the order $O\left(I^{2}\right)$ for I→0, so the reproduction number $R_{c}$ is equal to $R_{c}$ in the model without any RCD. This means that RCD as modelled cannot interrupt sustained disease transmission, nor can it overcome the effect of importation as soon as local prevalence is too low. So either with or without importation, RCD can only affect the magnitude of the endemic equilibrium and not the threshold behaviour between endemic and disease-free equilibrium. The intuition behind this phenomenon is that, as prevalence decreases, both the number of detected index cases and the number of cases found in each investigation decrease, such that the number of cases found by RCD is reduced. In practice, it could happen that this effect is compensated by another one, such as an increased clustering of cases around index cases represented with unbounded targeting ratio τ. For example, following Chitnis et al. (2019), τ could also be a time-varying quantity, which depends on the prevalence in the population and the number of secondary cases investigated, such that the targeting ratio increases as prevalence decreases and as ν decreases. A parametric function of the prevalence and ν fitted to data from Zambia is presented in Chitnis et al. (2019) and further used in Reiker et al. (2019) and could be substituted to the fixed values of τ. In all cases, extinction events may also occur when the endemic equilibrium is very low and the model is simulated in the stochastic framework (cf. below).

#### 2.2.1 Relation between the transmission rate and observable quantities

Because reactive case detection is an intervention that modifies the way cases are detected and reported, the definition of reported incidence needs to be modified in consequence. Let us therefore introduce the following additional notations:

• $h_{1}=\rho \left(\lambda I^{*}+\delta \right)\left(1-I^{*}\right)+\rho fS_{L}^{*}$ is the incidence of directly-detected infections
• $h_{2}=\rho_{2}\iota^{*}\nu \tau \eta \left(U_{0}^{*}+U_{L}^{*}\right)$ is the incidence of reactively-detected infections, with $\iota^{*}$, $U_{0}^{*}$ and $U_{L}^{*}$ being the equilibrium values for ι, $U_{0}$ and $U_{L}$ respectively. The parameter $\rho_{2}$ is the reporting rate for reactively-detected infections.
• $h=h_{1}+h_{2}$ is the total incidence of detected cases

With a similar methodology to Champagne et al. (2022), the transmission rate can be related via a polynomial equation to observable quantities h, p and $h_{1}$ using the model’s equilibrium, as detailed in Appendix C. Therefore, in order to use this relationship in practice, the value for $h_{1}$ is required, in addition to h and p.

Two situations can arise in practice:

1. only the total number of new cases h is known, regardless of whether cases are detected via direct detection or via reactive detection. $h_{1}$ needs to be calculated.
2. the numbers of directly and reactively detected and non- reactively detected cases are both known ($h_{1}$ and $h_{2}$).

#### 2.2.2 Only the total number of new cases is known

In this situation, we need to calculate the value of $h_{1}$, and the value of $h_{2}$ will be $h_{2}=h-h_{1}$.

At equilibrium, adding the equations for $U_{L}$ and $U_{0}$ in (2), we obtain $U_{L}^{*}+U_{0}^{*}=h_{1}\frac{1-\alpha }{\rho \left(r+\iota^{*}\nu \tau \eta \right)}$ (see Appendix C1). We have $\iota^{*}=\min\left(\iota_{max},\rho \left(\lambda I^{*}+\delta \right)\left(1-I^{*}\right)+\rho fS_{L}^{*}\right)=\min\left(\iota_{max},h_{1}\right)$, so we can evaluate separately the two possibilities.

If $\iota^{*}=\iota_{max}$, we have $\left(U_{L}^{*}+U_{0}^{*}\right)=\frac{\left(1-\alpha \right)h_{1}}{\rho \left(r+\iota_{max}\nu \tau \eta \right)}$. Hence $h=h_{1}+\iota_{max}\rho_{2}\nu \tau \eta \left(U_{L}^{*}+U_{0}^{*}\right)=h_{1}+\frac{\iota_{max}\rho_{2}\nu \tau \eta \left(1-\alpha \right)}{\rho \left(r+\iota_{max}\nu \tau \eta \right)}h_{1}$ and

$$h_{1}=\frac{h\rho \left(r+\iota_{max}\nu \tau \eta \right)}{\rho r+\iota_{max}\nu \tau \eta \left(\rho +\left(1-\alpha \right)\rho_{2}\right)}.$$

If $\iota^{*}=h_{1}$, we have $\left(U_{L}^{*}+U_{0}^{*}\right)=\frac{\left(1-\alpha \right)h_{1}}{\rho \left(r+h_{1}\nu \tau \eta \right)}$. Then, from $h=h_{1}+h_{1}\rho_{2}\nu \tau \eta \left(U_{L}^{*}+U_{0}^{*}\right)=h_{1}+\frac{\rho_{2}\nu \tau \eta \left(1-\alpha \right)}{\rho \left(r+h_{1}\nu \tau \eta \right)}h_{1}^{2}$, we get

$$h_{1}=\frac{\rho \nu \tau \eta h-\rho r+\sqrt{{\left(\rho \nu \tau \eta h-\rho r\right)}^{2}+4\rho rh\left(\rho +\left(1-\alpha \right)\rho_{2}\right)\nu \tau \eta }}{2\left(\rho +\left(1-\alpha \right)\rho_{2}\right)\nu \tau \eta }.$$

Combining these two results, in the case of capped ι, we find

$$h_{1}=\left\{\begin{array}{cc} \frac{\rho \nu \tau \eta h-\rho r+\sqrt{{\left(\rho \nu \tau \eta h-\rho r\right)}^{2}+4\rho rh\left(\rho +\left(1-\alpha \right)\rho_{2}\right)\nu \tau \eta }}{2\left(\rho +\left(1-\alpha \right)\rho_{2}\right)\nu \tau \eta } & \text{if this is less than}\iota_{max}\\ \frac{h\rho \left(r+\iota_{max}\nu \tau \eta \right)}{\rho r+\iota_{max}\nu \tau \eta \left(\rho +\left(1-\alpha \right)\rho_{2}\right)} & \text{otherwise}. \end{array}\right)$$

#### 2.2.3 The numbers of reactively detected and non-reactively detected cases are both known

If we know both $h_{1}$ and $h_{2}$, we can calculate λ directly. Additionally, we can combine this information to get an estimate of τ, which is otherwise difficult to find. We will consider only the cases where $h_{2}>0$ (otherwise, there is no effect of RCD and therefore no reason for using $h_{2}$ to calculate τ).

At equilibrium, adding the equations for $U_{L}$ and $U_{0}$ in (2), we obtain

$$\left(U_{L}^{*}+U_{0}^{*}\right)=\frac{1}{r}\left[\frac{\left(1-\alpha \right)h_{1}}{\rho }-\frac{h_{2}}{\rho_{2}}\right],$$

which can be used in

$$\tau =\frac{h_{2}}{\rho_{2}\iota^{*}\nu \eta \left(U_{L}^{*}+U_{0}^{*}\right)}.$$

### 2.3 Including Mass Drug Administration (MDA, model 3)

**Fig. 3:**
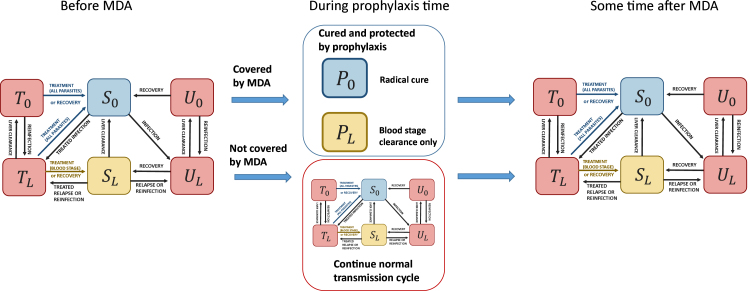
Schematic representation of the MDA model (model 3). Additional compartments $P_{L}$ and $P_{0}$ depict individuals protected by the drug prophylaxis. The subscripts L and 0 denote respectively the presence and absence of liver-stage parasites. In red are depicted the infectious compartments, with effective treatment access ($T_{0}$ and $T_{L}$) and without ($U_{0}$ and $U_{L}$). $S_{0}$ indicates malaria-free individuals while $S_{L}$ corresponds to individuals without blood-stage infection but with latent liver-stage parasites.

In order to model the deployment of an MDA campaign, the state variables representing the infectious population are depleted at the time of the MDA deployment depending on the MDA coverage. In order to model the time during which drug prophylaxis prevents targeted individuals from reinfections following MDA deployment, another model including two additional compartments is used. Finally, at the end of the prophylaxis time, the initial model without MDA can be simulated from the newly found initial condition. The overall framework is presented graphically in Fig. 3.

We present here the equations of the model without RCD as an example. Other model options with RCD follow the same approach and are detailed in Appendix D.

The simulation starts with the model defined by (1). MDA deployment is then modulated via four parameters, namely the time of MDA deployment $t_{MDA}$, the duration of prophylaxis $p_{MDA}$, the effective coverage $c_{MDA}$ and the percentage of radical cure $\beta_{MDA}$. The effective coverage $c_{MDA}$ and the percentage of radical cure $\beta_{MDA}$ can be informed by a decision tree similar to the ones of α and β for routine treatment. In particular, if MDA is implemented with 8-aminoquinolines, $\beta_{MDA}$ should reflect the expected average proportion of positive G6PD testing and if MDA is implemented without 8-aminoquinolines $\beta_{MDA}=0$. All four MDA parameters do not come into play in the ODEs. Instead, they affect the state variables of the system at a fixed point in time. At the time of MDA deployment $t_{MDA}$, the state variables of the system are modified as follows (using the notation $f\left(t_{MDA}^{-}\right)$ for the limit of f(t) as t approaches $t_{MDA}$):

(3) $$U_{L}\left(t_{MDA}\right)=\left(1-c_{MDA}\right)U_{L}\left(t_{MDA}^{-}\right)$$

$$U_{0}\left(t_{MDA}\right)=\left(1-c_{MDA}\right)U_{0}\left(t_{MDA}^{-}\right)$$

$$T_{L}\left(t_{MDA}\right)=\left(1-c_{MDA}\right)T_{L}\left(t_{MDA}^{-}\right)$$

$$T_{0}\left(t_{MDA}\right)=\left(1-c_{MDA}\right)T_{0}\left(t_{MDA}^{-}\right)$$

$$S_{L}\left(t_{MDA}\right)=\left(1-c_{MDA}\right)S_{L}\left(t_{MDA}^{-}\right)$$

$$S_{0}\left(t_{MDA}\right)=\left(1-c_{MDA}\right)S_{0}\left(t_{MDA}^{-}\right)$$

$$P_{L}\left(t_{MDA}\right)=c_{MDA}\left(1-\beta_{MDA}\right)\left(T_{L}\left(t_{MDA}^{-}\right)+U_{L}\left(t_{MDA}^{-}\right)+S_{L}\left(t_{MDA}^{-}\right)\right)$$

$$P_{0}\left(t_{MDA}\right)=c_{MDA}\beta_{MDA}\left(T_{L}\left(t_{MDA}^{-}\right)+U_{L}\left(t_{MDA}^{-}\right)+S_{L}\left(t_{MDA}^{-}\right)\right)$$

$$\;+c_{MDA}\left(T_{0}\left(t_{MDA}^{-}\right)+U_{0}\left(t_{MDA}^{-}\right)+S_{0}\left(t_{MDA}^{-}\right)\right).$$

To model the two effects of MDA (treatment and prophylaxis), we include two new compartments in the model. Let $P_{L}$ and $P_{0}$ be the proportions of people that are effectively covered by MDA with and without liver-stage infection, respectively. We have the following set of ODEs to describe the dynamics of the system during the prophylaxis period:

(4) $$\frac{dU_{L}}{dt}=\left(1-\alpha \right)\left(\lambda I+\delta \right)\left(1-I\right)+\left(1-\alpha \right)fS_{L}+\left(\lambda I+\delta \right)U_{0}-\gamma_{L}U_{L}-rU_{L}$$

$$\frac{dU_{0}}{dt}=-\left(\lambda I+\delta \right)U_{0}+\gamma_{L}U_{L}-rU_{0}$$

$$\frac{dT_{L}}{dt}=\alpha \left(\lambda I+\delta \right)\left(1-I\right)+\alpha fS_{L}+\left(\lambda I+\delta \right)T_{0}-\gamma_{L}T_{L}-\left(r+\sigma \right)T_{L}$$

$$\frac{dT_{0}}{dt}=-\left(\lambda I+\delta \right)T_{0}+\gamma_{L}T_{L}-\left(r+\sigma \right)T_{0}$$

$$\frac{dS_{L}}{dt}=-\left(\lambda I+\delta +f\right)S_{L}+\left(1-\beta \right)\sigma T_{L}-\gamma_{L}S_{L}+r\left(T_{L}+U_{L}\right)$$

$$\frac{dS_{0}}{dt}=-\left(\lambda I+\delta \right)S_{0}+\beta \sigma T_{L}+\sigma T_{0}+\gamma_{L}S_{L}+r\left(T_{0}+U_{0}\right)$$

$$\frac{dP_{L}}{dt}=-\gamma_{L}P_{L}$$

$$\frac{dP_{0}}{dt}=\gamma_{L}P_{L}$$

starting with the initial conditions defined in (3). We are assuming constant level of protection over time during the prophylaxis period, i.e. no decay in drug effectiveness in (4). Each individual either has perfect prophylaxis during the intervention or none at all in which case they go through the usual infection pathway.

Finally, at time $t_{MDA}+p_{MDA}$, the state variables are updated as follows:

$$U_{L}\left(t_{MDA}+p_{MDA}\right)=U_{L}\left({\left(t_{MDA}+p_{MDA}\right)}^{-}\right)$$

$$U_{0}\left(t_{MDA}+p_{MDA}\right)=U_{0}\left({\left(t_{MDA}+p_{MDA}\right)}^{-}\right)$$

$$T_{L}\left(t_{MDA}+p_{MDA}\right)=T_{L}\left({\left(t_{MDA}+p_{MDA}\right)}^{-}\right)$$

$$T_{0}\left(t_{MDA}+p_{MDA}\right)=T_{0}\left({\left(t_{MDA}+p_{MDA}\right)}^{-}\right)$$

$$S_{L}\left(t_{MDA}+p_{MDA}\right)=S_{L}\left({\left(t_{MDA}+p_{MDA}\right)}^{-}\right)+P_{L}\left({\left(t_{MDA}+p_{MDA}\right)}^{-}\right)$$

$$S_{0}\left(t_{MDA}+p_{MDA}\right)=S_{0}\left({\left(t_{MDA}+p_{MDA}\right)}^{-}\right)+P_{0}\left({\left(t_{MDA}+p_{MDA}\right)}^{-}\right)$$

$$P_{L}\left(t_{MDA}+p_{MDA}\right)=0$$

$$P_{0}\left(t_{MDA}+p_{MDA}\right)=0$$

and the initial model (1) is further simulated from these initial conditions.

Several MDA rounds can be chained using the same approach.

### 2.4 Including vector control

As mentioned in Champagne et al. (2022), vector control can be included in the model as a reduction of the intensity of transmission λ, following Briët et al. (2019). The transmission parameter λ becomes ωλ where ω∈[0,1] represents the intensity of vector control. The absence of vector control corresponds to the case ω=1, and ω=0 represents perfect vector control that completely disables vector-borne transmission.

If vector control interventions are present at baseline, the back-calculation methodology provides an estimate of ωλ instead of λ. Hence, the estimated λ value needs to be divided by the baseline value of ω in all formulae, as it is implemented by default in the R package. If the value of ω at baseline cannot be estimated, then all future scenarios for the other interventions (treatments, RCD, MDA) need to be interpreted as “keeping the same level of vector control compared to baseline”.

The ω parameter can be informed quantitatively by an external model for vector dynamics, for example with the methodology by Briët et al. (2019). Assuming that mosquitoes have a shorter generation time than humans, one can consider vector dynamics to be at equilibrium and approximate parasite dynamics at low endemicity with a human-to-human transmission model, where the transmission parameter is proportional to the vectorial capacity (see equation 21 in Smith and McKenzie (2004)). Under such assumptions, reducing the vectorial capacity by x% would therefore be equivalent to reducing λ by x%; hence vectorial capacity reduction factors could be used to inform ω. In Briët et al. (2019), the *Anopheles* life-cycle model by Chitnis et al. (2008) is used at equilibrium to calculate reduction factors on the vectorial capacity when introducing vector control tools, such as insecticide-treated nets or indoor residual spraying. Golumbeanu et al. (2023) generalized this framework to any *Anopheles* vector and various vector control interventions, and provided a method for correcting impact predictions by the time spent indoors and outdoors by both hosts and vectors. The associated vectorial capacity reduction factors can be extracted for specific *Anopheles* species and insecticides using a dedicated R package (Golumbeanu et al., 2023).

By making ω time-varying, one can include a decay in vector control effectiveness, for example due to the waning of insecticide or the attrition of bednets, as in Briët et al., 2019, Briet et al., 2020 and Golumbeanu et al. (2023).

### 2.5 Model implementation

The *P. vivax* models are implemented in R (R Core Team, 2019) as a publicly available package (https://swisstph.github.io/VivaxModelR/). The package enables the user to calibrate and simulate all the model versions presented here (with and without delays in treatment effect, with and without RCD, with and without MDA). The package includes tests to ensure that the back-calculations and simulations with the various models are correctly implemented, as well as a user tutorial.

As explained in the tutorial vignette, the user first needs to provide baseline parameter values, as well as incidence and importation data for model calibration. These values should correspond to a stable intervention situation, for example the average over one or several years during which there was no important change in intervention policy. During this calibration step, the package provides the corresponding reproduction numbers and transmission level. Secondly, the user can simulate forward the model, modifying at wish the intervention parameters in the future. During this simulation step, the package provides the trajectories of all model compartments for the various intervention scenarios.

The ordinary differential equations solving relies on the *deSolve* package (Soetaert et al., 2021). When simulating future intervention scenarios with the model, all implementations offer the possibility for the importation rate δ and the vector control term ω to be time-varying, using any empirical function provided by the user as a dataset.

Thanks to the model’s simple compartmental structure, it can be simulated several times to propagate various sources of uncertainties, namely demographic stochasticity, data uncertainty and parameter uncertainty.

• Demographic stochasticity: a stochastic implementation with either Gillespie algorithm or τ-leap methodologies is provided relying on the *TiPS* package (Danesh et al., 2020, Danesh et al., 2021). Thanks to the convergence of the stochastic version of the model to its ODE counterpart for infinite population sizes (Kurtz, 1970), the back-calculation of the transmission rate obtained with the ODE steady-state can be used in the stochastic model and the coherence between the two model implementations is guaranteed. Such convergence is empirically verified as part of the set of automated tests included in the R package.
• Data uncertainty: because the relationship between the transmission rate λ and observed data on (h,p) is algebraic, any uncertainty distribution on (h,p) can be propagated, provided that a simulated sample of (h,p) values is available. An example of such sample is provided in Champagne et al. (2022), with Poisson and binomial distributions for reported incidence and the proportion of imported cases and it is implemented in the R package.
• Parameter uncertainty: when a range or distribution of parameter values is available, uncertainty can be propagated by simulating the model on each parameter set of the sample.

## 3 Application: an illustrative example

As an illustrative example, we simulate three fictitious areas with varying reported case numbers, assuming perennial *P. vivax* transmission and values similar to Panama’s endemic regions in 2018 modelled in Champagne et al. (2022), as indicated in Table 3. The number of reported cases in each area increases from very low in Area 1 to moderate-high in Area 3. In Area 2, it is assumed that there is no importation and all cases are contracted locally. Data uncertainty was propagated by taking 1000 samples of the joint distribution of local and imported cases, assuming Poisson and binomial distributions as in Champagne et al. (2022). Effective blood stage treatment is represented by a treatment pathway including the presence of asymptomatic infections, access to care and diagnosis sensitivity. Effective radical cure of liver-stage parasites is represented by a treatment pathway including primaquine efficacy and adherence for patients with and without Directly Observed Treatment (Champagne et al., 2022). G6PD testing is not included in the β parameter as it is not part of the treatment guidelines in Panama in 2018. All treated and reactively-detected cases are assumed to be reported in the observed data (ρ=α and $\rho_{2}=1$). The three areas are assumed to have the same intervention parameters at baseline for case management, reactive case detection and vector control. MDA being a transient intervention, it is assumed to be absent at baseline.

When calibrating the model at baseline with the chosen parameter values, malaria is sustained through importation in Area 1 as $R_{c}$ is smaller than 1 (cf. Table 3). On the contrary, Areas 2 and 3 experience sustained local transmission ($R_{c}>1$), a result that was expected by construction in Area 2 due to the assumed absence of importation.

**Fig. 4:**
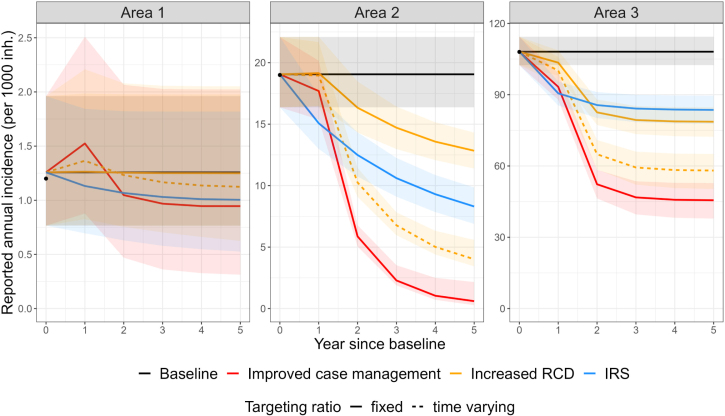
Effect of various intervention scenarios on *P. vivax* reported annual incidence, applied in three artificial settings. The parameter values corresponding to each scenario can be found in Table 4. The solid line represents the fixed-time median of all 1000 simulations. Uncertainty is represented in the shaded areas, which depict the 90% most central curves following the all-or-nothing approach mentioned in Juul et al. (2021) (sampling 50 curves, with 100 replications). The black dots represent the observed baseline annual incidence.

From there, five intervention scenarios are simulated, as detailed in Table 4. The first three interventions are case management strengthening, reactive case detection strengthening and deployment of indoor residual spraying (IRS, with Bendiocarb and 60% coverage) : the parameters corresponding to these interventions are detailed in Table 4 and their impact on reported annual incidence is presented in Fig. 4. In this example, case management strengthening has large impacts on malaria incidence. In the short term, an increase in reported incidence is observed in Area 1: this is due to the higher number of infections being reported when access to care is increased. In the long term, this reporting effect is counteracted by the effect of treatment reducing onward malaria transmission, and in all three settings, case management strengthening always leads to a large decrease in malaria incidence although it is not sufficient to reach elimination within the modelled timelines.

**Table 3:** Model parameters and data at baseline for three fictitious areas.

Baseline parameters	Area 1	Area 2	Area 3
**Setting-specific data**			
Reported cases per year	6	95	540
Imported cases per year	1	0	27
Population size	5000	5000	5000

**Setting-specific parameters at baseline**			
Access to care (a)	0.5	0.5	0.5
Proportion of infections effectively cured for blood stage (α)	a∗s∗d	a∗s∗d	a∗s∗d
Radical cure among treated infections (β)	0.86∗ϵ	0.86∗ϵ	0.86∗ϵ
Observation rate (ρ)	α	α	α
Average delay in treatment effect (1/σ, in days)	10	10	10
Vector control (ω, ω=1 corresponds to the absence of control)	1	1	1
RCD: max. index cases investigated (ι, per week per 5000 inh.)	50	50	50
RCD: number of individuals investigated per index case (ν)	5	5	5
RCD: targeting ratio (τ)	5	5	5
RCD: probability to detect and treat a case (η)	d	d	d
RCD: observation rate for RCD-detected cases ($\rho_{2}$)	1	1	1
MDA: coverage ($c_{MDA}$)	0	0	0
MDA: radical cure treatment ($\beta_{MDA}$)	0	0	0

**Fixed parameters**			
Relapse frequency (f, in days^−1^)	1/72 (White et al., 2016)		
Blood stage clearance rate (r, in days^−1^)	1/60 (White et al., 2016)		
Liver stage clearance rate ($\gamma_{L}$, in days^−1^)	1/223 (White et al., 2016)		
PQ efficacy (ϵ)	0.76 (Álvarez et al., 2006, Champagne et al., 2022)		
RDT sensitivity (d)	0.95 (Abba et al., 2014)		
Symptomatic contribution (s)	0.7, approximate value for Peru in Ferreira et al. (2022)		

**Estimated reproduction numbers**			
$R_{0}$	1.14	1.88	2.11
$R_{c}$	0.67	1.09	1.23

Reactive case detection has a much larger impact in areas with higher endemicity as there are more cases to be detected. In the three examples considered, using the time-varying targeting ratio from Chitnis et al. (2019) leads to stronger incidence reductions than the fixed value, as the targeting ratio increases with decreasing prevalence and hence accelerate the decrease in malaria trends. Similarly to the scenario with case management, in Area 1, a short-term increase in incidence is observed, due to the increased number of cases detected when strengthening the RCD intervention.

Finally, IRS leads to important reductions in malaria incidence, but is not sufficient to reduce transmission close to elimination levels. This is because, in this example, an important proportion of mosquito bites is not prevented by IRS, due to the early biting behaviour of *An. albimanus* and the assumption that individuals are outside and therefore unprotected before going to bed. Therefore, in this particular example, improvements in case managements have a greater impact on incidence compared to the introduction of IRS or the strengthening of RCD. In other settings with other parameter values, the conclusion might differ.

**Fig. 5:**
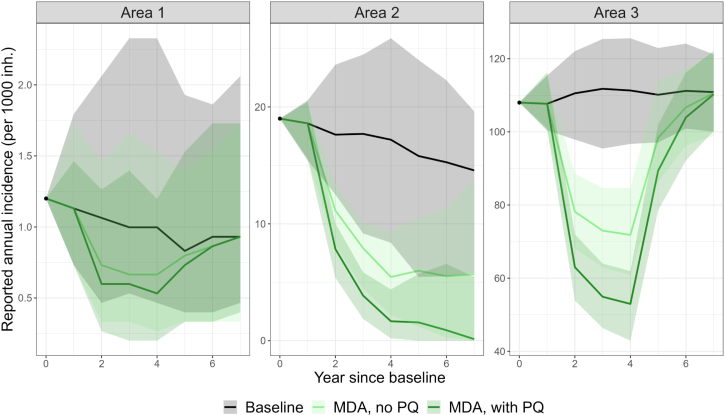
Effect of MDA scenarios on *P. vivax* reported annual incidence, applied in three artificial settings. The parameter values corresponding to each scenario can be found in Table 4. The solid line represents the fixed-time median of all 5000 simulations. Uncertainty is represented in the shaded areas, which depict the 90% most central curves following the all-or-nothing approach mentioned in Juul et al. (2021) (sampling 50 curves, with 100 replications). The black dots represent the observed baseline annual incidence.

The last two scenarios include MDA with and without PQ for radical cure (see Fig. 5). The effective MDA coverage for blood-stage treatment is assumed to be 80%. In the scenario with PQ MDA, it is assumed that only 50% of the individuals receiving effective MDA also receive effective radical cure treatment: many individuals receiving MDA might not be eligible to primaquine (due to age or G6PD status, for instance), might not adhere to the treatment scheme or might experience drug failure. The exact proportion could be adapted to represent most accurately the context of interest. Because of the high variability expected when the model reaches low case numbers, the stochastic version of the model is used, with 5000 independent simulation replicates. In this example, data uncertainty is not propagated, to focus on the stochasticity aspect. The effect of MDA is transient, and malaria transmission bounces back to initial prevalence levels when the intervention is interrupted. As expected, targeting the liver-stage reservoir with PQ increases the prevalence reduction achieved. In the scenario with very low transmission (Area 1), elimination is reached in only 6% of the simulations because transmission is sustained with importation, elimination being defined in this example as a whole year with zero cases (including reported and unreported). Other definitions of malaria elimination in the presence of importation, distinguishing local, indigenous and imported cases (Das et al., 2023) could also be explored but are beyond the scope of the current work. In the scenario without importation (Area 2), elimination in 7 years is reached in 28% and 48% of the simulations with MDA, without and with PQ respectively, highlighting the difficulty to reach this outcome.

**Table 4:** Parameter values of the intervention scenarios simulated.

Scenario	Description
Baseline	All parameters remain unchanged
Improved case management	Access to care is increased to 80% (α=0.8∗s∗d, ρ=0.8∗s∗d)
	Radical cure is increased to 90% (β=0.9∗ϵ)
	Delays in treatment are reduced (1/σ=5)
Increased RCD	The number of investigations per index case is increased (ν=10)
IRS	IRS with Bendiocarb is deployed every year with 60% coverage,
	for a vector with bionomics and biting rhythms comparable to
	*An. albimanus* (Briët et al., 2019) and human rhythms from Haiti (Knutson, 2014),
	assuming people are outside when not in bed as in Briët et al. (2019))
	(ω=0.56 at deployment, reverting gradually to 1 over a 6-month period,
	calculated as in Briët et al. (2019) with (Golumbeanu et al., 2023)).
MDA, no PQ	MDA is deployed in 3 rounds spaced by 1 year,
	with 30 days prophylaxis duration
	reaching 80% coverage ( $c_{MDA}=0.8$), without treatment for radical cure ($\beta_{MDA}=0$)
MDA, with PQ	MDA is deployed in 3 rounds spaced by 1 year
	with 30 days prophylaxis duration
	reaching 80% coverage ( $c_{MDA}=0.8$), with treatment for radical cure ($\beta_{MDA}=0.5$)

## 4 Discussion

In this work, we presented an extended compartmental model for *P. vivax* dynamics at the population level that includes four commonly deployed interventions, namely treatments, vector control, reactive case detection and mass drug administration. This model is suitable for low-endemicity settings without significant seasonal pattern. As we illustrate with fictitious data only, it can be calibrated in each setting of interest at the local level using the observed incidence at steady-state, thus providing estimates of transmission potential. The setting-specific model can then be used to compare the impact of various intervention strategies and identify the most promising ones. The model can be simulated in both the deterministic and the stochastic framework and it is readily implemented as a publicly available R package.

Thanks to its compartmental structure and the steady-state assumptions, the model is easy to apply to routinely collected *P. vivax* data on case counts and imported cases, with additional parameters informed by literature and knowledge on currently deployed interventions. The model thus has the potential to be used in a timely-manner for strategic planning questions within a country. Although the current manuscript does not provide a real-life application to demonstrate such uses, a previous version of the model was applied to district-level data from the four endemic regions of Panama (Champagne et al., 2022) and we include here an illustration with synthetic data. In this fictitious example, the effect of increasing case management levels, vector control coverage and reactive case detection intensity or deploying MDA are compared to identify what strategy would be most impactful for each area. In a real-life application, such results would then need to be contextualized with complementary knowledge on local specificities, operational feasibility, and intervention costs through iterative interactions with stakeholders, as in Runge et al. (2020).

The model incorporates the effect of routine malaria treatment on transmission through three parameters: the proportion of blood-stage infections that are effectively treated, the proportion of treated cases that achieve radical cure of the liver-stage parasites and the duration of infectivity for treated cases. These three aspects are important components of health system strengthening strategies and the possibility to compare improvements in these three dimensions simultaneously or independently can provide useful information for decision-makers. These parameters can be informed by a decision tree representing the treatment pathway for infected individuals, similar to that of Galactionova et al. (2015) or White et al. (2018) for example. The structure of this decision tree can be adapted to the local context: for instance, G6PD testing was not included in the simulated example presented here to match the treatment guidelines in Panama in 2018, but it would need to be included for any application in a setting where G6PD testing is required. The values within the decision tree can be informed by surveys on access to care practices, programmatic data on the health system and biological studies on drug efficacy or the presence of symptoms. Nonetheless, it is important to note that the model is very sensitive to the value of baseline case management parameters (Champagne et al., 2022) and the modelled impact of case management strategies relies heavily on the availability and quality of data at the spatial scale of interest.

The mathematical representation of RCD relies on the definition of a targeting ratio, and thus does not include mechanistically the effect of case clustering as would be the case in spatially-explicit individual-based models (Gerardin et al., 2017). Therefore, similarly to Chitnis et al., 2019, Reiker et al., 2019, Das et al., 2022 and Das et al. (2023), it is necessary to parameterize the targeting ratio using setting-specific data (Das et al., 2022), parasite spatial signature analyses such as Sandfort et al. (2023) or by choosing appropriate assumptions adapted to the local context. Epidemiological knowledge of infection risk factors to evaluate if such a strategy is suitable in a given setting is therefore considered as a prerequisite before using the model. For example, in countries where *P. vivax* infection is mainly driven by occupational exposure (e.g. working in the forest), the investigation of the neighbourhood of index cases might be less efficient compared to occupational screening (Mukaka et al., 2021): this effect could be represented with a smaller targeting ratio. This appreciation is therefore left to the user when choosing the appropriate value for targeting ratio in the setting of interest. Nonetheless, if data on the source of case reporting (via RCD or via other detection channels) is available, the value of the targeting ratio can be quantified at baseline using the back-calculation methodology presented here. The objective of the current model is therefore not to investigate if RCD has the capacity to detect cases in general, but rather to evaluate its impact in relation to other interventions and for various implementation designs.

In line with other modelling work (Robinson et al., 2015; Pemberton-Ross et al., 2017, White et al., 2018, Obadia et al., 2022) and with the available epidemiological evidence (Shah et al., 2021), MDA is represented as an instantaneous shock on the state variables without affecting the model parameters. Therefore, its effect is transient by construction and malaria dynamics are expected to revert to their previous equilibrium except in the stochastic case if elimination is reached, or if accompanied by sustainable changes such as increased intervention coverages or environmental modifications. Decay in the effectiveness of prophylaxis is not explicitly modelled, rather the model assumes a constant level of protection during the time of prophylaxis. This simplification should not strongly affect the annual results for drugs with prophylaxis duration of 15 to 30 days. Nonetheless, the model could also be extended to include parametric decay forms.

Mosquito dynamics are not directly included in the model and the effect of vector control is represented as a reduction of the human-to-human transmission intensity. This simplifying assumption is chosen because the time scale of vector dynamics is much shorter than the one of human dynamics, such that in a low endemicity setting where the proportion of infectious host is small, the parasite dynamics can be approximated by an SIS-like model (Smith and McKenzie, 2004). Additionally, using the model by Golumbeanu et al. (2023) enables the user to account for differences in vector control efficacy due to mosquito species biological characteristics, intervention type or the proportion of time that individuals spend indoors or in bed (Briët et al., 2019), whose importance can be crucial, especially in elimination settings outside of Africa.

Seasonality is not included in the model, therefore, it cannot be used to study the optimal timing of an intervention during the year or to evaluate the impact of interventions with short term efficacy in seasonal settings. It is only suitable for perennial settings or when studying annual data and interventions that do not have seasonal effects. Nonetheless, the R package implementation of the model offers the possibility to include a seasonal forcing into the transmission rate via the time-varying parameter ω when simulating disease dynamics over time. Adapting the analytical back-calculation methodology and reproduction number definition to the seasonal case (Bacaër, 2007) is however outside the scope of the current work. Instead, when adding a periodic seasonal-forcing in the model, simulation-based fitting methods should be used to calibrate the transmission rate.

Thanks to its stochastic implementation (Danesh et al., 2020, Danesh et al., 2021), the model can be used to compute elimination probabilities and timelines, which are useful outcomes to compare malaria intervention strategies in low-endemicity settings. Additionally, the inclusion of demographic stochasticity opens the possibility to use the model for small population sizes, as required to represent *P. vivax* dynamics at local scales. The simple model structure also allows the user to run the model several times in order to propagate uncertainties in data sources and parameters, as illustrated with case observation uncertainty in the first application example.

The model is a simplified representation of reality and has therefore a certain number of limitations. Firstly, the chosen representation of treatment delays assumes that host infectiousness to mosquitoes is constant over time, which is an important simplification in regard to the complex life cycle of the malaria parasites (Gaspoz et al., 2022) and the relaxation of this assumption will be the object of future work. In addition, delays in treatment effect were represented with exponential durations via the ODE formalism and not fixed durations as would have been the case with delay differential equations (Kim et al., 2021). Nonetheless, the chosen formalism of the model presents some similarities with a model based on delayed-differential equations, as illustrated in Appendix A for a simpler model with perfect radical cure.

Moreover, because of its emphasis on implementation simplicity, this model makes some additional simplifications in the biological depiction of *P. vivax*. Importantly, the model does not include any form of immunity, an assumption which is only acceptable in settings with low to moderate transmission level, as in Champagne et al. (2022). Specific effects of the multiplicity of infection on the duration of infection (Henry, 2020, Anwar et al., 2023a) are also ignored, assuming that such situations occur rarely in low-endemicity settings. Relapses are modelled to occur at a constant rate, based on White et al. (2016) which relied on White et al. (2014), although the underlying mechanism could be represented with increased details (Mehra et al., 2022). The model excludes the possibility for successful radical cure without blood-stage clearance, as this situation is very rare in practice because eligibility criteria, access and adherence are particularly restricted for liver stage treatment (Thriemer et al., 2021). This compartmental model also relies on various homogeneity assumptions, as differences in infectiousness or susceptibility related to severity levels, exposure to mosquito bites or age groups are not represented, thus ignoring the potential impact of such heterogeneities on disease dynamics (Stadler et al., 2022). Nonetheless, the model can be applied to data at local scale, such as districts or localities, in order to explore the effects of geographical heterogeneities. Moreover, previous work (Champagne et al., 2022) compared model predictions on changes in treatment strategies with a more complex individual model (Nekkab et al., 2021) and found comparable results in low to moderate endemicity settings.

Finally, the back-calculation methodology for baseline calibration relies on the steady state of the deterministic model: as discussed in Champagne et al. (2022), this makes the assumption that transmission is stable over the years and that transmission would remain at the same constant level in the absence of any intervention changes. This assumption may not hold when countries reach very low incidence levels where transmission is sporadic, and other modelling approaches such as individual reproduction numbers (Routledge et al., 2018) should be used instead. Nonetheless, equilibrium assumptions are commonly used in applied malaria models (see for example Nekkab et al., 2021, White et al., 2018 and Sherrard-Smith et al. (2022)), because it provides a clearly defined baseline counterfactual scenario. If temporal data is available and a clear temporal trend is observed that cannot be mechanistically explained by intervention or structural changes, other statistical methods for time series fitting of compartmental models could be used instead (Chatzilena et al., 2019, FitzJohn et al., 2021).

Despite these limitations, this model provides a useful additional tool that could be used to support country-specific decision making in the choice of interventions to deploy in various areas. Thanks to its analytical foundations and its simplicity of implementation, we believe that this model has the potential to support decision making on malaria strategies in a rapid and transparent manner.

## CRediT authorship contribution statement

**C. Champagne:** Conceptualization, Methodology, Software, Formal analysis, Investigation, Writing – original draft. **M. Gerhards:** Conceptualization, Methodology, Formal analysis, Investigation, Writing – original draft. **J.T. Lana:** Conceptualization, Investigation, Writing – review & editing. **A. Le Menach:** Conceptualization, Writing – review & editing, Project administration, Funding acquisition. **E. Pothin:** Conceptualization, Methodology, Writing – review & editing, Project administration, Funding acquisition.

## Declaration of competing interest

None.

## Data Availability

The computer code utilized in this manuscript is available at: https://github.com/SwissTPH/VivaxModelR.

## Appendix A Supplementary data

MMC S1 Additional equations and proofs: rationale for model 1’s structure, transmission rate calculation in model 1 and model 2 and RCD-MDA models.
