# Supplementary material for: Quantifying the impact of interventions against Plasmodium vivax: A model for country-specific use
Source: Epidemics. 2024 Mar;46:100747. doi: 10.1016/j.epidem.2024.100747 (PMC10944169; doi:10.1016/j.epidem.2024.100747)
Supplement: MMC S1 — Additional equations and proofs: rationale for model 1’s structure, transmission rate calculation in model 1 and model 2 and RCD-MDA models. [file mmc1.pdf]

Quantifying the impact of interventions against *Plasmodium vivax*: a model for country-specific use - Appendix -

C. Champagne[1,2,*,**], M. Gerhards[1,2,*], J. Lana[3], A. Le Menach[3] and E. Pothin[1,2,3]

[1]Swiss Tropical and Public Health Institute, Basel, Switzerland
[2]University of Basel, Basel, Switzerland
[3]Clinton Health Access Initiative, Boston, USA
[*]equal contribution
[**]Corresponding author: clara.champagne@swisstph.ch

Contents

A Including delay in treatment effect: rationale for the chosen model **2**
A.1 Delay with constant duration: using delay differential equations 2
A.2 Delay with exponential waiting time: using ordinary differential equations 3

B Back calculation of the transmission rate for model 1 **5**
B.1 Model equilibrium 5
B.2 Polynomial equation 6
B.3 Relationship with observable quantities 7

C Back calculation of the transmission rate for RCD models **10**
C.1 RCD model (model 2) 10
C.2 RCD model with referral to health facility (model 2b) 12

D Models with MDA and RCD **14**
D.1 MDA-RCD model (model 3a) 14
D.2 MDA-RCD model with referral to health facility (model 3b) 15

A Including delay in treatment effect: rationale for the chosen model

The model without delay in treatment effect [Champagne et al., 2022] is defined by the following system of ordinary differential equations (model 0):

$$\begin{aligned}
\frac{dI_L}{dt} &= (1-\alpha)(\lambda(I_L+I_0)+\delta)(S_0+S_L) + (\lambda(I_L+I_0)+\delta)I_0 + (1-\alpha)fS_L - \gamma_L I_L - rI_L \\
\frac{dI_0}{dt} &= -(\lambda(I_L+I_0)+\delta)I_0 + \gamma_L I_L - rI_0 \\
\frac{dS_L}{dt} &= -(1-\alpha(1-\beta))(\lambda(I_L+I_0)+\delta+f)S_L + \alpha(1-\beta)(\lambda(I_0+I_L)+\delta)S_0 - \gamma_L S_L + rI_L \\
\frac{dS_0}{dt} &= -(1-\alpha\beta)(\lambda(I_L+I_0)+\delta)S_0 + (\lambda(I_0+I_L)+\delta)(\alpha\beta S_L) + \alpha\beta f S_L + \gamma_L S_L + rI_0
\end{aligned}$$

In this model, the effect of treatment is instantaneous, however, in reality individuals do not get treated instantaneously. Therefore, we aim to modify the model to include some time delay between infection and clearance of the parasite for treated individuals. We define $I := 1 - S_L - S_0$ the proportion of individuals with blood-stage infections and will focus on the case with $\beta = 1$ (perfect radical cure for treated individuals).

Following the approach of Arino and Van den Driessche [Arino and van den Driessche, 2006] (vaccination model), we have the following equation for I:

$$I(t) = I^{Start}(t) + \int_0^t \big[(\lambda I(u)+\delta)(1-I(u)) + fS_L(u)\big] P(t-u) e^{-(t-u)r} du \tag{5}$$

where $P(t-u)$ is the proportion of those who were infected at time u and have not been effectively treated at time t. Two choices for the function P are explored in the following sections: the first one represents a delay with constant duration and the second one a delay with geometric duration.

A.1 Delay with constant duration: using delay differential equations

If effective treatment is applied to a proportion α after a fixed time σ^{-1}, the function P is a step function

$$P(t) = \begin{cases} 1 & \text{for } t < \sigma^{-1} \\ 1-\alpha & \text{for } t \geq \sigma^{-1} \end{cases}.$$

Inserting in the equation (5) we obtain the following equation:

$$\begin{aligned}
I(t) = & I^{Start}(t) + \int_{t-\sigma^{-1}}^{t} \big[(\lambda I(u)+\delta)(1-I(u)) + fS_L(u)\big] e^{-(t-u)r} du \\
& + (1-\alpha)\int_0^{t-\sigma^{-1}} \big[(\lambda(I(u)+\delta)(1-I(u)) + fS_L(u)\big] e^{-(t-u)r} du
\end{aligned}$$

Differentiation yields the following delay differential equation:

$$\begin{aligned}
\frac{dI}{dt} = & (\lambda I(t)+\delta)(1-I(t)) + fS_L(t) \\
& - \alpha e^{-r/\sigma}\big[(\lambda I(t-\sigma^{-1})+\delta)(1-I(t-\sigma^{-1})) + fS_L(t-\sigma^{-1})\big] \\
& - r\big[I(t) - I^{Start}(t)\big] + \frac{dI^{Start}(t)}{dt}
\end{aligned}$$

A.2 Delay with exponential waiting time: using ordinary differential equations

If effective treatment is applied to a proportion α after a exponentially distributed waiting time with mean σ^{-1}, the function P is of the form

$$P(t) = (1-\alpha) + \alpha e^{-\sigma t}.$$

If we assume this treatment scheme to apply to those starting being infected, the equation 5 reduces to

$$\begin{aligned}
I(t) =&(1-\alpha)I(0)e^{-rt} + \alpha_{corr}I(0)e^{-(r+\sigma)t} \\
&+ (1-\alpha)\int_0^t \big[(\lambda I(u)+\delta)(1-I(u)) + fS_L(u)\big]e^{r(t-u)}du \\
&+ \alpha\int_0^t \big[(\lambda I(u)+\delta)(1-I(u)) + fS_L(u)\big]e^{(\sigma+r)(t-u)}du.
\end{aligned}$$

Differentiation yields

$$\begin{aligned}
\frac{dI}{dt} =& -r(1-\alpha)I(0)e^{-rt} - (r+\sigma)\alpha I(0)e^{-(r+\sigma)t} \\
&+ (1-\alpha)\big[(\lambda I(t)+\delta)(1-I(t)) + fS_L(t)\big] - r(1-\alpha)\int_0^t \big[(\lambda I(u)+\delta)(1-I(u)) + fS_L(u)\big]e^{-r(t-u)}du \\
&+ \alpha\big[(\lambda I(t)+\delta)(1-I(t)) + fS_L(t)\big] - (r+\sigma)\alpha\int_0^t \big[(\lambda I(u)+\delta)(1-I(u)) + fS_L(u)\big]e^{-(r+\sigma)(t-u)}du.
\end{aligned}$$

We can simplify this by separating those α that get effective treatment from those $1-\alpha$ that don't. If we consider this separation not at the time the treatment is effective but at the time of infection, this defines two separate classes U for those that don't have access to treatment and T for those that have access to treatment into which α of the newly infected get sorted. Of these, $\frac{\sigma}{r+\sigma}$ actually get effective treatment, after an average waiting time of $\frac{1}{r+\sigma}$, accounting for the possibility to recover before receiving the effective treatment. Then, we have a system of integral equations

$$\begin{aligned}
U_L(t) + U_0(t) =&(U_L(0) + U_0(0))e^{-rt} + \int_0^t (1-\alpha)\big[(\lambda I(u)+\delta)(1-I(u)) + fS_L(u)\big]e^{-r(t-u)}du \\
T_L(t) + T_0(t) =&(T_L(0) + T_0(0))e^{-(r+\sigma)t} + \int_0^t \alpha\big[(\lambda I(u)+\delta)(1-I(u)) + fS_L(u)\big]e^{-(r+\sigma)(t-u)}du
\end{aligned}$$

which when differentiated yield

$$\begin{aligned}
\frac{d(U_L+U_0)}{dt}(t) =&(1-\alpha)\big[(\lambda I(t)+\delta)(1-I(t)) + fS_L(t)\big] - r(U_L(t)+U_0(t)) \\
\frac{d(T_L+T_0)}{dt}(t) =&\alpha\big[(\lambda I(t)+\delta)(1-I(t)) + fS_L(t)\big] - (r+\sigma)(T_L(t)+T_0(t))
\end{aligned}$$

and therefore to

$$\begin{aligned}
\frac{dU_L}{dt} &= (1-\alpha)(\lambda(U_L+U_0+T_L+T_0)+\delta)(S_0+S_L) + (\lambda(U_L+U_0+T_L+T_0)+\delta)U_0 + (1-\alpha)fS_L - \gamma_L U_L - rU_L \\
\frac{dU_0}{dt} &= -(\lambda(U_L+U_0+T_L+T_0)+\delta)U_0 + \gamma_L U_L - rU_0 \\
\frac{dS_L}{dt} &= (1-\beta)\sigma T_L - (\lambda(U_0+U_L+T_L+T_0)+\delta)S_L - fS_L - \gamma_L S_L + rU_L + rT_L \\
\frac{dS_0}{dt} &= -(\lambda(U_L+U_0+T_L+T_0)+\delta)S_0 + \sigma T_L + \sigma T_0 + \gamma_L S_L + rU_0 + rT_0 \\
\frac{dT_L}{dt} &= \alpha(\lambda(U_L+U_0+T_L+T_0)+\delta)(S_0+S_L) + \alpha fS_L - \sigma T_L - rT_L - \gamma_L T_L + (\lambda(U_L+U_0+T_L+T_0)+\delta)T_0 \\
\frac{dT_0}{dt} &= \gamma_L T_L - \sigma T_0 - (\lambda(U_L+U_0+T_L+T_0)+\delta)T_0 - rT_0
\end{aligned} \tag{6}$$

which is equivalent to model 1 in the main text for the case $\beta = 1$.
As a final remark, one can note that taking the limit $\sigma \to \infty$ we get the initial model with instantantaneous treatment (model 0), while letting $\sigma = 0$ we get model 0 without treatment, as is to be expected.

It is worth noting that the actual probability of getting effectively treated in both scenarios is not α (since some infected will already have recovered by the end of the delay), but $\alpha_{corr_d} = \alpha e^{-r\sigma}$ in the model with with constant delay and $\alpha_{corr} = \alpha \frac{\sigma}{r+\sigma}$ in the model with geometrically distributed delay. These numbers are also the proportions by which the average length of infection is reduced by including treatment.

These results are not easily generalized to the case where $\beta < 1$, due to the complexity of the integral equations. Nonetheless, the model in equation (6) was extended to the case with imperfect radical cure ($\beta < 1$) leading to model 1 presented in the main text.

B Back calculation of the transmission rate for model 1

B.1 Model equilibrium

Let T_L^*, T_0^*, U_L^*, U_0^*, S_L^*, S_0^*, and I^* be the equilibrium proportions. As $r > 0$ is verified in all biologically plausible cases, we will continue with this assumption throughout the rest of the paper.

At the equilibrium, we have the equations

$$0 = \frac{dU_L}{dt} = (1-\alpha)(\lambda I^* + \delta)(1 - I^*) + (1-\alpha) f S_L^* + (\lambda I^* + \delta) U_0^* - \gamma_L U_L^* - r U_L^* \tag{7}$$

$$0 = \frac{dU_0}{dt} = -(\lambda I^* + \delta) U_0^* + \gamma_L U_L^* - r U_0^* \tag{8}$$

$$0 = \frac{dT_L}{dt} = \alpha(\lambda I^* + \delta)(1 - I^*) + \alpha f S_L^* + (\lambda I^* + \delta) T_0^* - \gamma_L T_L^* - (r+\sigma) T_L^* \tag{9}$$

$$0 = \frac{dT_0}{dt} = -(\lambda I^* + \delta) T_0^* + \gamma_L T_L^* - (r+\sigma) T_0^* \tag{10}$$

$$0 = \frac{dS_L}{dt} = -(\lambda I^* + \delta + f) S_L^* + (1-\beta)\sigma T_L^* - \gamma_L S_L^* + r(T_L^* + U_L^*) \tag{11}$$

$$0 = \frac{dS_0}{dt} = -(\lambda I^* + \delta) S_0^* + \beta\sigma T_L^* + \sigma T_0^* + \gamma_L S_L^* + r(T_0^* + U_0^*) \tag{12}$$

By adding equations (9) and (10) we obtain the additional equation:

$$0 = \alpha(\lambda I^* + \delta)(1 - I^*) + \alpha f S_L^* - (r+\sigma)(T_L^* + T_0^*)$$

Likewise, by adding equations (7) and (8) we obtain the further equation:

$$0 = (1-\alpha)(\lambda I^* + \delta)(1 - I^*) + (1-\alpha) f S_L^* - r(U_0^* + U_L^*).$$

Hence we find the relations

$$\begin{aligned} T_L^* + T_0^* &= \frac{\alpha}{r+\sigma}\big((\lambda I^* + \delta)(1 - I^*) + f S_L^*\big), \\ U_L^* + U_0^* &= \frac{1-\alpha}{r}\big((\lambda I^* + \delta)(1 - I^*) + f S_L^*\big), \end{aligned}$$

which can be combined to get

$$I^* = T_L^* + T_0^* + U_0^* + U_L^* = \frac{r + (1-\alpha)\sigma}{r(r+\sigma)}\big((\lambda I^* + \delta)(1 - I^*) + f S_L^*\big) \tag{13}$$

and

$$\begin{aligned} T_L^* + T_0^* &= \frac{\alpha r}{r + (1-\alpha)\sigma} I^* \\ U_L^* + U_0^* &= \frac{(1-\alpha)(r+\sigma)}{r + (1-\alpha)\sigma} I^* \end{aligned}$$

We start by solving equation (10) for T_0^*:

$$\begin{aligned} T_0^* &= \frac{\gamma_L}{\lambda I^* + \delta + \gamma_L + r + \sigma}(T_L^* + T_0^*) \\ &= \frac{\gamma_L}{\lambda I^* + \delta + \gamma_L + r + \sigma} \frac{\alpha r}{r + (1-\alpha)\sigma} I^*, \end{aligned}$$

from which we arrive at the equation

$$T_L^* = \frac{\lambda I^* + \delta + r + \sigma}{\lambda I^* + \delta + \gamma_L + r + \sigma} \frac{\alpha r}{r + (1-\alpha)\sigma} I^*,$$

(neither of the denominators is zero as we assumed $r > 0$).
Likewise, we solve equation (8) for U_0^*:

$$U_0^* = \frac{\gamma_L}{\lambda I^* + \delta + \gamma_L + r}(U_0^* + U_L^*)$$
$$= \frac{\gamma_L}{\lambda I^* + \delta + \gamma_L + r}\frac{(1-\alpha)(r+\sigma)}{r+(1-\alpha)\sigma}I^*,$$

from which we arrive at the equation

$$U_L^* = \frac{\lambda I^* + \delta + r}{\lambda I^* + \delta + \gamma_L + r}\frac{(1-\alpha)(r+\sigma)}{r+(1-\alpha)\sigma}I^*,$$

(again, neither of the denominators is zero).
Solving (11) for S_L^* gives:

$$S_L^* = \frac{rU_L^* + (r + (1-\beta)\sigma)T_L^*}{\lambda I^* + \delta + \gamma_L + f} \tag{14}$$

(the denominator cannot be 0, since $\lambda I^* = \delta = f = 0$ would imply $h = 0$, see below).

Plugging both the equations for T_L^* and U_L^* into (14) yields:

$$S_L^* = \frac{r(1-\alpha)(r+\sigma)(\lambda I^* + \delta + r)(\lambda I^* + \delta + \gamma_L + r + \sigma)I^* + (r + (1-\beta)\sigma)\alpha r(\lambda I^* + \delta + r + \sigma)(\lambda I^* + \delta + \gamma_L + r)I^*}{(\lambda I^* + \delta + \gamma_L + f)(\lambda I^* + \delta + \gamma_L + r)(\lambda I^* + \delta + \gamma_L + r + \sigma)(r + (1-\alpha)\sigma)}$$
$$= \frac{(r + (1-\alpha\beta)\sigma)(\lambda I^* + \delta + r)(\lambda I^* + \delta + \gamma_L + r + \sigma) + \alpha(r + (1-\beta)\sigma)\gamma_L\sigma}{(\lambda I^* + \delta + \gamma_L + f)(\lambda I^* + \delta + \gamma_L + r)(\lambda I^* + \delta + \gamma_L + r + \sigma)(r + (1-\alpha)\sigma)}rI^*$$

We can simplify further by using the identity $\alpha_{corr} = \alpha\frac{\sigma}{r+\sigma}$:

$$S_L^* = \frac{(1-\alpha_{corr}\beta)(\lambda I^* + \delta + r)(\lambda I^* + \delta + \gamma_L + r + \sigma) + \alpha_{corr}(r + (1-\beta)\sigma)\gamma_L}{(\lambda I^* + \delta + \gamma_L + f)(\lambda I^* + \delta + \gamma_L + r)(\lambda I^* + \delta + \gamma_L + r + \sigma)(1-\alpha_{corr})}rI^*.$$

B.2 Polynomial equation

Now, plugging this into (13) and multiplying by $(\lambda I^* + \delta + \gamma_L + f)(\lambda I^* + \delta + \gamma_L + r)(\lambda I^* + \delta + \gamma_L + r + \sigma)$ we obtain:

$$0 =(\lambda I^* + \delta + \gamma_L + f)(\lambda I^* + \delta + \gamma_L + r)(\lambda I^* + \delta + \gamma_L + r + \sigma)\left((\lambda I^* + \delta)(1 - I^*) - \frac{r(r+\sigma)I^*}{r+(1-\alpha)\sigma}\right)$$
$$+ \frac{rI^*}{r+(1-\alpha)\sigma}f(r + (1-\alpha\beta)\sigma)(\lambda I^* + \delta + r)(\lambda I^* + \delta + \gamma_L + r + \sigma) + \frac{rI^*}{r+(1-\alpha)\sigma}f\alpha(r + (1-\beta)\sigma)\gamma_L\sigma \tag{15}$$

or, equivalently,

$$0 =(\lambda I^* + \delta + \gamma_L + f)(\lambda I^* + \delta + \gamma_L + r)(\lambda I^* + \delta + \gamma_L + r + \sigma)\left((\lambda I^* + \delta)(1 - I^*) - \frac{rI^*}{1-\alpha_{corr}}\right)$$
$$+ \frac{rI^*}{1-\alpha_{corr}}f(1-\alpha_{corr}\beta)(\lambda I^* + \delta + r)(\lambda I^* + \delta + \gamma_L + r + \sigma) + \frac{rI^*}{1-\alpha_{corr}}f\alpha_{corr}(r + (1-\beta)\sigma)\gamma_L$$

Rearranging the terms by powers of λ, we get the equation:

$$
\begin{aligned}
0 =&\lambda^4 I^{*4}(1-I^*)\\
&+\lambda^3 I^{*3}\left[(1-I^*)(4\delta+3\gamma_L+2r+f+\sigma)-\frac{rI^*}{1-\alpha_{corr}}\right]\\
&+\lambda^2 I^{*2}\Bigg[(1-I^*)\big((\delta+\gamma_L+r)(3\delta+3\gamma_L+r+2f)+\delta(3\delta+3\gamma_L+2r+f)+\sigma(3\delta+2\gamma_L+r+f)\big)\\
&\quad-\frac{rI^*}{1-\alpha_{corr}}(3\delta+3\gamma_L+2r+\alpha_{corr}\beta f+\sigma)\Bigg]\\
&+\lambda I^*\Bigg[(1-I^*)(\delta+\gamma_L+r)\big[(\delta+\gamma_L+f)(\delta+\gamma_L+r)+\delta(3\delta+3\gamma_L+r+2f)\big]\\
&\quad+(1-I^*)\sigma\big[(\delta+\gamma_L+f)(\delta+\gamma_l+r)+\delta(2\delta+2\gamma_L+r+f)\big]\\
&\quad-\frac{rI^*}{1-\alpha_{corr}}\big[(\delta+\gamma_L+r)(3\delta+3\gamma_L+r+2\alpha_{corr}\beta f)+(1-\alpha_{corr}\beta)f\gamma_L\big]\\
&\quad-\frac{rI^*}{1-\alpha_{corr}}\sigma(2\delta+2\gamma_L+r+\alpha_{corr}\beta f)\Bigg]\\
&+(1-I^*)\delta(\delta+\gamma_L+r)(\delta+\gamma_L+f)(\delta+\gamma_L+r+\sigma)\\
&\quad-\frac{rI^*}{1-\alpha_{corr}}\Big[(\delta+\gamma_L+r)\big[(\delta+\gamma_L+r)(\delta+\gamma_L+\alpha_{corr}\beta f)+(1-\alpha_{corr}\beta)\gamma_L f\big]-\alpha_{corr}f\gamma_L r\Big]\\
&\quad-\frac{rI^*}{1-\alpha_{corr}}\sigma\big[(\delta+\gamma_L+r)(\delta+\gamma_L+\alpha_{corr}\beta f)+(1-\alpha_{corr})f\gamma_L\big]
\end{aligned}
$$

As long as the assumptions $r > 0$ and $h > 0$ are met, multiplication by the denominator of S_L^* is an equivalent transformation, so any non-negative root of this polynomial is a solution of the system of equilibrium equations.
It can be noted that the equation in [Champagne et al., 2022] for model 0 can be derived from this one by dividing by σ and taking the limit $\sigma \to \infty$ (corresponding to a delay decreasing to 0).

We get the same qualitative result as in the setting without delayed treatment (model 0 [Champagne et al., 2022]):

Theorem B.1. *If $h > 0$ and $r > 0$, the function*

$$
\begin{aligned}
P(\lambda) =&(\lambda I^*+\delta+\gamma_L+f)(\lambda I^*+\delta+\gamma_L+r)(\lambda I^*+\delta+\gamma_L+r+\sigma)\left((\lambda I^*+\delta)(1-I^*)-\frac{rI^*}{r+(1-\alpha)\sigma}(r+\sigma)\right)\\
&+\frac{rI^*}{r+(1-\alpha)\sigma}f(r+(1-\alpha\beta)\sigma)(\lambda I^*+\delta+r+\sigma)(\lambda I^*+\delta+\gamma_L+r)-\frac{rI^*}{r+(1-\alpha)\sigma}(1-\alpha)f(r+\sigma)\gamma_L\sigma
\end{aligned}
$$

has at most one positive real root.
It has two non-negative real roots (i.e. one of them is 0) only if $\alpha\beta\sigma = \gamma_L = \delta = 0$ (corresponding to an equilibrium of relapses and recoveries without liver-stage clearance).

B.3 Relationship with observable quantities

The observed incidence, i.e. the rate of observed newly arising blood-stage infections, was defined as $h := \rho[(\lambda I^* + \delta)(1 - I^*) + fS_L^*]$, where ρ is a reporting rate. Starting from equation (13), we can calculate I^* from observed quantities and model parameters as:

$$
I^* = \frac{h(r+(1-\alpha)\sigma)}{\rho r(r+\sigma)} \tag{16}
$$

(with $r > 0$ as assumed previously). If on the other hand $h = 0$, we have $I^* = 0$. Being in the disease-free equilibrium makes it impossible to derive λ. Because of this, we will also make the further assumption $h > 0$. It is worth noting that in this model, as opposed to that without treatment delay (model 0, [Champagne et al., 2022]), $\alpha = 1$ is not ruled out.

The proportion p of imported cases is defined such that $ph := \rho\delta(1 - I^*)$ represents the imported cases and $(1 - p)h = \rho[\lambda I^*(1 - I^*) + fS_L^*]$ the locally acquired cases. Therefore, δ can be derived from observed quantities and model parameters exactly as in the model without delay:

$$\delta = \frac{ph}{\rho(1 - I^*)} = \frac{phr(r + \sigma)}{\rho r(r + \sigma) - h(r + (1 - \alpha)\sigma)} \tag{17}$$

Thanks to these relationships, the values for I^* and δ can be plugged in the polynomial equation. Thanks to Theorem B.1, it is possible to solve this polynomial equation numerically in order to back-calculate the parameter λ required to reproduce the reported data, assuming that the model is at equilibirum.

Proof of Theorem B.1. Before beginning the proof, we point to the fact that $h > 0$ and $r > 0$ imply $0 < I^* < 1$. We start with the case $f = 0$ like in the model without delay (model 0 [Champagne et al., 2022]). From the shape of equation 15 one can easily see that the four roots in that case are

$$-\frac{\gamma_L + \delta + r + \sigma}{I^*}, -\frac{\gamma_L + \delta + r}{I^*}, \quad -\frac{\gamma_L + \delta}{I^*} \quad \text{and} \quad \frac{r(r + \sigma)}{r + (1 - \alpha)\sigma}\frac{1}{1 - I^*} - \frac{\delta}{I^*},$$

ordered from smallest to largest. Only the last two of them may be non-negative, and the first of these only in the case $\gamma_L = \delta = 0$, and in that case, from $\lambda = \delta = f = 0$ it follows $h = 0$, contradicting our assumption, so this is no solution.
Now we turn to the case $f > 0$.
To make the dependence of P on f visible, let us denote it P_f. Then,

$$P_f(\lambda) = P_0(\lambda) + fQ(\lambda)$$

with

$$\begin{aligned}
Q(\lambda) &= (\lambda I^* + \delta + \gamma_L + r)(\lambda I^* + \delta + \gamma_L + r + \sigma)\left((\lambda I^* + \delta)(1 - I^*) - \frac{r(r + \sigma)}{r + (1 - \alpha)\sigma}I^*\right) \\
&\quad + \frac{r(r + (1 - \alpha\beta)\sigma)}{r + (1 - \alpha_T)\sigma}I^*(\lambda I^* + \delta + r + \sigma)(\lambda I^* + \delta + \gamma_L + r) - \frac{r(r + \sigma)}{r + (1 - \alpha)\sigma}I^*(1 - \alpha)\gamma_L\sigma \\
&= \underbrace{(\lambda I^* + \delta + \gamma_L + r)(\lambda I^* + \delta + \gamma_L + r + \sigma)\left((\lambda I^* + \delta)(1 - I^*) - \frac{\alpha\beta r\sigma}{r + (1 - \alpha)\sigma}I^*\right)}_{R(\lambda)} \\
&\quad + \underbrace{\left(-\frac{r(r + (1 - \alpha\beta)\sigma)}{r + (1 - \alpha)\sigma}I^*\gamma_L(\lambda I^* + \delta + \gamma_L + r)\right) - \frac{r(r + \sigma)}{r + (1 - \alpha)\sigma}I^*(1 - \alpha)\gamma_L\sigma}_{S(\lambda)}
\end{aligned}$$

It is easily seen that $P_0 + fR$ is a polynomial of degree 4 with positive leading coefficient and two roots at $\lambda_1 := -\frac{\delta+\gamma_L+r+\sigma}{I^*}$ and $\lambda_2 := -\frac{\delta+\gamma_L+r}{I^*}$.
Let us now first consider the case $\gamma_L > 0$. In that case we also see that

$$\underbrace{P_0(-\frac{\delta}{I^*})}_{<0} + \underbrace{fR(-\frac{\delta}{I^*})}_{\leq 0} < 0.$$

As $\lim_{\lambda\to\infty} P_0(\lambda) + fR(\lambda) = \infty$, from the intermediate value theorem it follows that $P_0 + fR$ has a root λ_4 to the right of $-\frac{\delta}{I^*}$. Since every simple root is accompanied with a sign change, the last root λ_3 has to be to the left of $-\frac{\delta}{I^*}$. In particular, λ_1, λ_2 and λ_3 are all negative.
We will now prove that by adding $fS(\lambda) = -f\frac{r(r+(1-\alpha\beta)\sigma)}{r+(1-\alpha)\sigma}I^*\gamma_L(\lambda I^* + \delta + \gamma_L + r) - f\frac{r(r+\sigma)}{r+(1-\alpha)\sigma}I^*(1 - \alpha)$, we will not get a non-negative root:
It is easily seen that $fS(\lambda) < 0$ for all $\lambda \geq 0$, so

$$P_f(\lambda) = P_0(\lambda) + fR(\lambda) + fS(\lambda) < 0 \quad \text{for } \lambda \in [0, \lambda_4],$$

implying that there cannot be a root in that interval.
On the other hand, it is a known fact that the inflection points of a polynomial of degree 4 lie between the smallest and the largest root. This implies that $P_0 + fR$ is strictly convex in the interval $]\lambda_4, \infty[$. Since $P_f = P_0 + fR + fS$ has the same second derivative, it is also strictly convex in that interval. This, combined with the fact that $P_f(\lambda_4) = fS(\lambda_4) < 0$, implies that there is only one root of P_f in that interval. This finishes the proof in the case $\gamma_L > 0$.

Now we turn to the case $\gamma_L = 0$. Then, $P_f = P_0 + fR$.
We again know the two roots $-\frac{\delta+r+\sigma}{I^*}$, $-\frac{\delta+r}{I^*}$ and we see that

$$P_f(-\frac{\delta}{I^*}) = \underbrace{P_0(-\frac{\delta}{I^*})}_{=0} + fR(-\frac{\delta}{I^*}) = -\underbrace{fr(r+\sigma)}_{\neq 0}\frac{\alpha\beta r\sigma}{r+(1-\alpha)\sigma}I^*.$$

If this term is 0 (which is the case if and only if $\alpha\beta\sigma = 0$), we know that $-\frac{\delta}{I^*}$ is also a root of P_f. This root is only non-negative if $\delta = 0$, which is the case of endless relapses and recoveries without liver-stage clearance.
Otherwise, the term must be negative. Then again, since every simple root is accompanied with a sign change, we know that there must be exactly one root to the right of $-\frac{\delta}{I^*}$. □

C Back calculation of the transmission rate for RCD models

C.1 RCD model (model 2)

In this section, we provide the results for the calibration of the RCD model (model 2) from the main text.

C.1.1 Model equilibrium

At equilibrium, adding the equations for U_L and U_0 in (2), we get

$$0 = (1-\alpha)(\lambda I^* + \delta)(1 - I^*) + (1-\alpha) f S_L^* - (r + \iota^* \nu\tau\eta)(U_L^* + U_0^*) \tag{18}$$

and similarly by adding the equations for T_L and T_0 in (2),

$$0 = \alpha(\lambda I^* + \delta)(1 - I^*) + \alpha f S_L^* - (r + \sigma)(T_L^* + T_0^*) \tag{19}$$

Finally, by adding both of these, we find

$$(\lambda I^* + \delta)(1 - I^*) + f S_L^* = (r + \sigma)(T_0^* + T_L^*) + (r + \iota^* \nu\tau\eta)(U_L^* + U_0^*) \tag{20}$$

which can be inserted into either (18) or (19) to get

$$(1-\alpha)(r+\sigma)(T_0^* + T_L^*) = \alpha(r + \iota^*\nu\tau\eta)(U_0^* + U_L^*) \tag{21}$$

$$U_L^* + U_0^* = \frac{(1-\alpha)(r+\sigma)}{r + (1-\alpha)\sigma + \alpha\iota^*\nu\tau\eta} I^* \tag{22}$$

$$T_L^* + T_0^* = \frac{\alpha(r + \iota^*\nu\tau\eta)}{r + (1-\alpha)\sigma + \alpha\iota^*\nu\tau\eta} I^* \tag{23}$$

From the equation for U_0 in (2), we obtain

$$\begin{aligned} U_0^* &= \frac{\gamma_L (U_L^* + U_0^*)}{\lambda I^* + \delta + \gamma_L + r + \iota^*\nu\tau\eta} \\ &= \frac{\gamma_L (1-\alpha)(r+\sigma)}{\lambda I^* + \delta + \gamma_L + r + \iota^*\nu\tau\eta} \frac{I^*}{r + (1-\alpha)\sigma + \alpha\iota^*\nu\tau\eta} \\ U_L^* &= \frac{(\lambda I^* + \delta + r + \iota^*\nu\tau\eta)(1-\alpha)(r+\sigma)}{\lambda I^* + \delta + \gamma_L + r + \iota^*\nu\tau\eta} \frac{I^*}{r + (1-\alpha)\sigma + \alpha\iota^*\nu\tau\eta}. \end{aligned}$$

From the equation for T_0 in (2), we obtain

$$\begin{aligned} T_0^* &= \frac{\gamma_L (T_0^* + T_L^*)}{\lambda I^* + \delta + \gamma_L + r + \sigma} \\ &= \frac{\gamma_L \alpha (r + \iota^*\nu\tau\eta)}{\lambda I^* + \delta + \gamma_L + r + \sigma} \frac{I^*}{r + (1-\alpha)\sigma + \alpha\iota^*\nu\tau\eta} \\ T_L^* &= \frac{(\lambda I^* + \delta + r + \sigma)\alpha(r + \iota^*\nu\tau\eta)}{\lambda I^* + \delta + \gamma_L + r + \sigma} \frac{I^*}{r + (1-\alpha)\sigma + \alpha\iota^*\nu\tau\eta}. \end{aligned}$$

Inserting these results into the equation for S_L in (2), yields

$$S_L^* = \frac{(r + (1-\beta)\iota^*\nu\tau\eta)U_L^* + (r + (1-\beta)\sigma)T_L^*}{\lambda I^* + \delta + \gamma_L + f}$$

C.1.2 Polynomial equation

Plugging this into (20) yields

$$
\begin{aligned}
0 =& P_{RCD}(\lambda) \\
=& (\lambda I^* + \delta + \gamma_L + f)(\lambda I^* + \delta + \gamma_L + r + \iota^*\nu\tau\eta)(\lambda I^* + \delta + \gamma_L + r + \sigma)\left((1 - I^*)(\lambda I^* + \delta) - \frac{(r + \iota^*\nu\tau\eta)(r + \sigma)I^*}{r + (1-\alpha)\sigma + \alpha\iota^*\nu\tau\eta}\right) \\
&+ \frac{f\big(r^2 + (1-\alpha\beta)r\sigma + (1-(1-\alpha)\beta)r\iota^*\nu\tau\eta + (1-\beta)\sigma\iota^*\nu\tau\eta\big)(\lambda I^* + \delta + r + \sigma)(\lambda I^* + \delta + \gamma_L + r + \iota^*\nu\tau\eta)I^*}{r + (1-\alpha)\sigma + \alpha\iota^*\nu\tau\eta} \\
&+ \frac{f(1-\alpha)(r + (1-\beta)\iota^*\nu\tau\eta)(r+\sigma)\gamma_L(\iota^*\nu\tau\eta - \sigma)I^*}{r + (1-\alpha_{corr})\sigma + \alpha\iota^*\nu\tau\eta} \\
=& P_{\tilde{r}=r+\iota^*\nu\tau\eta, \tilde{\sigma}=\sigma-\iota^*\nu\tau\eta}(\lambda) \\
&- \beta\iota^*\nu\tau\eta f(\lambda I^* + \delta + \tilde{r})(\lambda I^* + \delta + \gamma_L + \tilde{r} + \tilde{\sigma})I^* \\
&- \beta\iota^*\nu\tau\eta f \frac{\alpha\tilde{r}\tilde{\sigma}\gamma_L I^*}{\tilde{r} + (1-\alpha)\tilde{\sigma}}
\end{aligned}
$$

C.1.3 Relationship with observable quantities

From equation (18), we can derive

$$U_L^* + U_0^* = \frac{1-\alpha}{\rho(r + \iota^*\nu\eta\tau)} h_1 \tag{24}$$

Pluggin this into equation (22), we obtain

$$I^* = h_1 \frac{r + (1-\alpha)\sigma + \alpha\iota^*\nu\eta\tau}{\rho(r + \iota^*\nu\eta\tau)(r+\sigma)}$$

Finally, as in the model without RCD (model 1), we have also

$$\delta = \frac{ph}{\rho(1 - I^*)}$$

Therefore, the polynomial equation can be solved numerically, using the values for model parameters and observable quantities h, p and h_1.

C.1.4 Relation to other models

Model 2 is related to other models in the following way if ι is fixed instead of capped (i.e. if $\iota = \iota_{max}$):

- If $\beta = 0$ and $\sigma > \iota\nu\tau\eta$, the model is equivalent to the model without RCD (model 1) with r replaced with $r + \iota\nu\tau\eta$ and σ replaced with $\sigma - \iota\nu\tau\eta$.
- If $\beta = 0$ and $\sigma < \iota\nu\tau\eta$, the model is instead equivalent to the model without RCD (model 1) with r replaced with $r + \sigma$, σ replaced with $\iota\nu\tau\eta - \sigma$, α replaced with $1 - \alpha$ and T_L and T_0 swapped with U_L and U_0, respectively.
- If $\beta = 0$ and $\sigma = \iota\nu\tau\eta$, the model is equivalent to the model by [Champagne et al., 2022] (model 0) with r replaced with $r + \sigma$, α set to 0 and T_L and T_0 added to U_L and U_0, respectively.
- If $\sigma = 0$, the model is equivalent to the model without RCD (model 1) with σ replaced with $\iota\nu\tau\eta$, α replaced with $1 - \alpha$ and T_L and T_0 swapped with U_L and U_0, respectively.

Such correspondences are used in the R package to test the correctness of the model implementation.

C.2 RCD model with referral to health facility (model 2b)

We present here an alternative RCD model in which cases detected via RCD need to be referred to a health facility before being treated and therefore experience a delay in treatment effect. It is defined as follows (noting $I = U_L + U_0 + T_L + T_0$):

$$\begin{aligned}
\frac{dU_L}{dt} =&(1-\alpha)(\lambda I+\delta)(1-I) + (1-\alpha) f S_L + (\lambda I + \delta)U_0 - \gamma_L U_L - rU_L \\
&- \min(\iota_{max}, \rho(\lambda I+\delta)(1-I) + \rho f S_L)\nu\tau\eta U_L \\
\frac{dU_0}{dt} =& -(\lambda I+\delta)U_0 + \gamma_L U_L - rU_0 - \min(\iota_{max}, \rho(\lambda I+\delta)(1-I) + \rho f S_L)\nu\tau\eta U_0 \\
\frac{dT_L}{dt} =&\alpha(\lambda I+\delta)(1-I) + \alpha f S_L + (\lambda I+\delta)T_0 - \gamma_L T_L - (r+\sigma)T_L \\
&+ \min(\iota_{max}, \rho(\lambda I+\delta)(1-I) + \rho f S_L)\nu\tau\eta U_L \\
\frac{dT_0}{dt} =& -(\lambda I+\delta)T_0 + \gamma_L T_L - (r+\sigma)T_0 \\
&+ \min(\iota_{max}, \rho(\lambda I+\delta)(1-I) + \rho f S_L)\nu\tau\eta U_0 \\
\frac{dS_L}{dt} =& -(\lambda I^* + \delta + f)S_L + (1-\beta)\sigma T_L - \gamma_L S_L + r(T_L + U_L) \\
\frac{dS_0}{dt} =& -(\lambda I+\delta)S_0 + \beta\sigma T_L + \sigma T_0 + \gamma_L S_L + r(T_0 + U_0)
\end{aligned} \tag{25}$$

Similarly to model 2, if there is no importation ($\delta = 0$) and τ is fixed (or at least bounded), all of the 'RCD terms' are of the order $O(I^2)$ for $I \to 0$, so the R_c values are the same and equal to the R_c without any RCD.

C.2.1 Model equilibrium

Adding the equations for U_L and U_0 in (25), we get

$$0 = (1-\alpha)(\lambda I^* + \delta)(1-I^*) + (1-\alpha) f S_L^* - r(U_L^* + U_0^*) - \iota^*\nu\tau\eta(U_L^* + U_0^*), \tag{26}$$

and similarly by adding the equations for T_L and T_0 in (25),

$$0 = \alpha(\lambda I^* + \delta)(1-I^*) + \alpha f S_L^* - (r+\sigma)(T_L^* + T_0^*) + \iota^*\nu\tau\eta(U_L^* + U_0^*). \tag{27}$$

Finally, by adding both of these, we find

$$(\lambda I^* + \delta)(1-I^*) + f S_L^* = (r+\sigma)(T_0^* + T_L^*) + r(U_L^* + U_0^*), \tag{28}$$

which can be inserted into either (26) or (27) to get

$$(1-\alpha)(r+\sigma)(T_0^* + T_L^*) = (\alpha r + \iota^*\nu\tau\eta)(U_L^* + U_0^*) \tag{29}$$

$$U_L^* + U_0^* = \frac{(1-\alpha)(r+\sigma)}{r + (1-\alpha)\sigma + \iota^*\nu\tau\eta} I^* \tag{30}$$

$$T_L^* + T_0^* = \frac{\alpha r + \iota^*\nu\tau\eta}{r + (1-\alpha)\sigma + \iota^*\nu\tau\eta} I^* \tag{31}$$

From the equation for U_0 in (25), we obtain

$$\begin{aligned}
U_0^* &= \frac{\gamma_L(U_L^* + U_0^*)}{\lambda I^* + \delta + \gamma_L + r + \iota^*\nu\tau\eta} \\
U_L^* &= (U_0^* + U_L^*) - U_0^*
\end{aligned}$$

From the equation for T_0 in (25), we obtain

$$T_0^* = \frac{\gamma_L(T_0^* + T_L^*) + \iota^*\nu\tau\eta U_0^*}{\lambda I^* + \delta + \gamma_L + r + \sigma}$$
$$T_L^* = (T_0^* + T_L^*) - T_0^*$$

Inserting these results into the equation for S_L in (25) yields

$$\begin{aligned} S_L^* =& \frac{rU_L^* + (r + (1-\beta)\sigma)T_L^*}{\lambda I^* + \delta + \gamma_L + f} \\ =& \frac{\big(r(r + (1-\alpha\beta)\sigma) + \iota^*\nu\tau\eta(r + (1-\beta)\sigma)\big)(\lambda I^* + \delta + r + \sigma)(\lambda I^* + \delta + \gamma_L + r + \iota^*\nu\tau\eta)I^*}{(\lambda I^* + \delta + \gamma_L + f)(\lambda I^* + \delta + \gamma_L + r + \iota^*\nu\tau\eta)(\lambda I^* + \delta + \gamma_L + r + \sigma)(r + (1-\alpha)\sigma + \iota^*\nu\tau\eta)} \\ &- \frac{(1-\alpha)(r + (1-\beta)\iota^*\nu\tau\eta)(r + \sigma)\gamma_L\sigma I^*}{(\lambda I^* + \delta + \gamma_L + f)(\lambda I^* + \delta + \gamma_L + r + \iota^*\nu\tau\eta)(\lambda I^* + \delta + \gamma_L + r + \sigma)(r + (1-\alpha)\sigma + \iota^*\nu\tau\eta)}. \end{aligned}$$

C.2.2 Polynomial equation

Plugging this into (28) yields

$$\begin{aligned} 0 =& P_{RCD_2}(\lambda) \\ =& (\lambda I^* + \delta + \gamma_L + f)(\lambda I^* + \delta + \gamma_L + r + \iota^*\nu\tau\eta)(\lambda I^* + \delta + \gamma_L + r + \sigma)\left((1 - I^*)(\lambda I^* + \delta) - \frac{(r + \iota^*\nu\tau\eta)(r+\sigma)I^*}{r + (1-\alpha)\sigma + \iota^*\nu\tau\eta}\right) \\ &+ \frac{I^*}{r + (1-\alpha)\sigma + \iota^*\nu\tau\eta} f\big(r(r + (1-\alpha\beta)\sigma) + \iota^*\nu\tau\eta(r + (1-\beta)\sigma)\big)(\lambda I^* + \delta + r + \sigma)(\lambda I^* + \delta + \gamma_L + r + \iota^*\nu\tau\eta) \\ &- \frac{I^*}{r + (1-\alpha)\sigma + \iota^*\nu\tau\eta} f(1-\alpha)(r + (1-\beta)\iota^*\nu\tau\eta)(r+\sigma)\gamma_L\sigma \end{aligned}$$

C.2.3 Relationship with observable quantities

From equation (26), we can derive

$$U_L^* + U_0^* = \frac{1-\alpha}{\rho(r + \iota^*\nu\eta\tau)} h_1$$

Pluggin this into equation (30), we obtain

$$I^* = h_1 \frac{r + (1-\alpha)\sigma + \iota^*\nu\eta\tau}{\rho(r + \iota^*\nu\eta\tau)(r+\sigma)}$$

Finally, as in the model without RCD (model 1), we have also

$$\delta = \frac{ph}{\rho(1 - I^*)}$$

Therefore, the polynomial equation can be solved numerically, using the values for model parameters and observable quantities h, p and h_1.

C.2.4 Relation to other models

This model is related to other models in the following way:

- If $\iota\nu\tau\eta = 0$, this model is the same as the model without RCD (model 1).
- If $\sigma = 0$, the model is equivalent to the model in [Champagne et al., 2022] (model 0) with T_L and T_0 added to U_L and U_0, respectively.

D Models with MDA and RCD

D.1 MDA-RCD model (model 3a)

The model is represented by the following ODE system:

$$\begin{aligned}
\frac{dU_L}{dt} &= (1-\alpha)(\lambda I+\delta)(1-I) + (1-\alpha)fS_L + (\lambda I+\delta)U_0 - \gamma_L U_L - rU_L \\
&\quad - \min(\iota_{max}, \rho(\lambda I+\delta)(1-I) + \rho f S_L)\nu\tau\eta U_L \\
\frac{dU_0}{dt} &= -(\lambda I+\delta)U_0 + \gamma_L U_L - rU_0 - \min(\iota_{max}, \rho(\lambda I+\delta)(1-I) + \rho f S_L)\nu\tau\eta U_0 \\
\frac{dS_L}{dt} &= -(\lambda I+\delta+f)S_L + (1-\beta)\sigma T_L - \gamma_L S_L + r(T_L+U_L) \\
&\quad + (1-\beta)\min(\iota_{max}, \rho(\lambda I+\delta)(1-I) + \rho f S_L)\nu\tau\eta U_L \\
\frac{dT_L}{dt} &= \alpha(\lambda I+\delta)(1-I) + \alpha f S_L + (\lambda I+\delta)T_0 - \gamma_L T_L - (r+\sigma)T_L \\
\frac{dT_0}{dt} &= -(\lambda I+\delta)T_0 + \gamma_L T_L - (r+\sigma)T_0 \\
\frac{dS_0}{dt} &= -(\lambda I+\delta)S_0 + \beta\sigma T_L + \sigma T_0 + \gamma_L S_L + r(T_0+U_0) \\
&\quad + \min(\iota_{max}, \rho(\lambda I+\delta)(1-I) + \rho f S_L)\nu\tau\eta(\beta U_L + U_0) \\
\frac{dP_L}{dt} &= -\gamma_L P_L \\
\frac{dP_0}{dt} &= \gamma_L P_L,
\end{aligned}$$

starting with $P_L(0) = P_0(0) = 0$.
At time t_{MDA}, we take the new values

$$\begin{aligned}
U_L(t_{MDA}) &= (1-c_{MDA})U_L(t_{MDA}^-) \\
U_0(t_{MDA}) &= (1-c_{MDA})U_0(t_{MDA}^-) \\
T_L(t_{MDA}) &= (1-c_{MDA})T_L(t_{MDA}^-) \\
T_0(t_{MDA}) &= (1-c_{MDA})T_0(t_{MDA}^-) \\
S_L(t_{MDA}) &= (1-c_{MDA})S_L(t_{MDA}^-) \\
S_0(t_{MDA}) &= (1-c_{MDA})S_0(t_{MDA}^-) \\
P_L(t_{MDA}) &= c_{MDA}(1-\beta_{MDA})\left(T_L(t_{MDA}^-) + U_L(t_{MDA}^-) + S_L(t_{MDA}^-)\right) \\
P_0(t_{MDA}) &= c_{MDA}\beta_{MDA}\left(T_L(t_{MDA}^-) + U_L(t_{MDA}^-) + S_L(t_{MDA}^-)\right) + c_{MDA}\left(T_0(t_{MDA}^-) + U_0(t_{MDA}^-) + S_0(t_{MDA}^-)\right).
\end{aligned}$$

At last, at time $t_{MDA} + p_{MDA}$, we take the new values

$$\begin{aligned}
U_L(t_{MDA}+p_{MDA}) &= U_L((t_{MDA}+p_{MDA})^-) \\
U_0(t_{MDA}+p_{MDA}) &= U_0((t_{MDA}+p_{MDA})^-) \\
T_L(t_{MDA}+p_{MDA}) &= T_L((t_{MDA}+p_{MDA})^-) \\
T_0(t_{MDA}+p_{MDA}) &= T_0((t_{MDA}+p_{MDA})^-) \\
S_L(t_{MDA}+p_{MDA}) &= S_L((t_{MDA}+p_{MDA})^-) + P_L((t_{MDA}+p_{MDA})^-) \\
S_0(t_{MDA}+p_{MDA}) &= S_0((t_{MDA}+p_{MDA})^-) + P_0((t_{MDA}+p_{MDA})^-) \\
P_L(t_{MDA}+p_{MDA}) &= 0 \\
P_0(t_{MDA}+p_{MDA}) &= 0.
\end{aligned}$$

D.2 MDA-RCD model with referral to health facility (model 3b)

The model is represented by the following ODE system:

$$\begin{aligned}
\frac{dU_L}{dt} &= (1-\alpha)(\lambda I+\delta)(1-I) + (1-\alpha)fS_L + (\lambda I+\delta)U_0 - \gamma_L U_L - rU_L \\
&\quad - \min(\iota_{max}, \rho(\lambda I+\delta)(1-I) + \rho f S_L)\nu\tau\eta U_L \\
\frac{dU_0}{dt} &= -(\lambda I+\delta)U_0 + \gamma_L U_L - rU_0 - \min(\iota_{max}, \rho(\lambda I+\delta)(1-I) + \rho f S_L)\nu\tau\eta U_0 \\
\frac{dT_L}{dt} &= \alpha(\lambda I+\delta)(1-I) + \alpha f S_L + (\lambda I+\delta)T_0 - \gamma_L T_L - (r+\sigma)T_L \\
&\quad + \min(\iota_{max}, \rho(\lambda I+\delta)(1-I) + \rho f S_L)\nu\tau\eta U_L \\
\frac{dT_0}{dt} &= -(\lambda I+\delta)T_0 + \gamma_L T_L - (r+\sigma)T_0 + \min(\iota_{max}, \rho(\lambda I+\delta)(1-I) + \rho f S_L)\nu\tau\eta U_0 \\
\frac{dS_L}{dt} &= -(\lambda I+\delta+f)S_L + (1-\beta)\sigma T_L - \gamma_L S_L + r(T_L+U_L) \\
\frac{dS_0}{dt} &= -(\lambda I+\delta)S_0 + \beta\sigma T_L + \sigma T_0 + \gamma_L S_L + r(T_0+U_0) \\
\frac{dP_L}{dt} &= -\gamma_L P_L \\
\frac{dP_0}{dt} &= \gamma_L P_L,
\end{aligned}$$

starting with $P_L(0) = P_0(0) = 0$.
At time t_{MDA}, we take the new values

$$\begin{aligned}
U_L(t_{MDA}) &= (1-c_{MDA})U_L(t_{MDA}^-) \\
U_0(t_{MDA}) &= (1-c_{MDA})U_0(t_{MDA}^-) \\
T_L(t_{MDA}) &= (1-c_{MDA})T_L(t_{MDA}^-) \\
T_0(t_{MDA}) &= (1-c_{MDA})T_0(t_{MDA}^-) \\
S_L(t_{MDA}) &= (1-c_{MDA})S_L(t_{MDA}^-) \\
S_0(t_{MDA}) &= (1-c_{MDA})S_0(t_{MDA}^-) \\
P_L(t_{MDA}) &= c_{MDA}(1-\beta_{MDA})\left(T_L(t_{MDA}^-) + U_L(t_{MDA}^-) + S_L(t_{MDA}^-)\right) \\
P_0(t_{MDA}) &= c_{MDA}\beta_{MDA}\left(T_L(t_{MDA}^-) + U_L(t_{MDA}^-) + S_L(t_{MDA}^-)\right) + c_{MDA}\left(T_0(t_{MDA}^-) + U_0(t_{MDA}^-) + S_0(t_{MDA}^-)\right).
\end{aligned}$$

At last, at time $t_{MDA} + p_{MDA}$, we take the new values

$$\begin{aligned}
U_L(t_{MDA}+p_{MDA}) &= U_L((t_{MDA}+p_{MDA})^-) \\
U_0(t_{MDA}+p_{MDA}) &= U_0((t_{MDA}+p_{MDA})^-) \\
T_L(t_{MDA}+p_{MDA}) &= T_L((t_{MDA}+p_{MDA})^-) \\
T_0(t_{MDA}+p_{MDA}) &= T_0((t_{MDA}+p_{MDA})^-) \\
S_L(t_{MDA}+p_{MDA}) &= S_L((t_{MDA}+p_{MDA})^-) + P_L((t_{MDA}+p_{MDA})^-) \\
S_0(t_{MDA}+p_{MDA}) &= S_0((t_{MDA}+p_{MDA})^-) + P_0((t_{MDA}+p_{MDA})^-) \\
P_L(t_{MDA}+p_{MDA}) &= 0 \\
P_0(t_{MDA}+p_{MDA}) &= 0.
\end{aligned}$$

References

J. Arino and P. van den Driessche. Time delays in epidemic models: modeling and numerical considerations. In *Delay Differential Equations and Applications*. Springer Verlag, 2006.

C. Champagne, M. Gerhards, J. Lana, B. G. Espinosa, C. Bradley, O. González, J. M. Cohen, A. Le Menach, M. T. White, and E. Pothin. Using observed incidence to calibrate the transmission level of a mathematical model for Plasmodium vivax dynamics including case management and importation. *Mathematical Biosciences*, page 108750, Jan. 2022. ISSN 0025-5564. doi: 10.1016/j.mbs.2021.108750. URL https://www.sciencedirect.com/science/article/pii/S0025556421001541.
